# A Randomised Placebo-Controlled Trial to Differentiate the Acute Cognitive and Mood Effects of Chlorogenic Acid from Decaffeinated Coffee

**DOI:** 10.1371/journal.pone.0082897

**Published:** 2013-12-09

**Authors:** David A. Camfield, Beata Y. Silber, Andrew B. Scholey, Karen Nolidin, Antionette Goh, Con Stough

**Affiliations:** 1 Centre for Human Psychopharmacology, Swinburne University, Melbourne, Australia; 2 Nestlé Research Centre, Lausanne, Switzerland; California Pacific Medicial Center Research Institute, United States of America

## Abstract

In the current study, sixty healthy older adults aged 50 years or older, and who were light to moderate coffee drinkers, were administered 6g of a decaffeinated green coffee blend (NESCAFÉ Green Blend coffee; GB) or 540mg pure chlorogenic acids (CGA) or placebo in a double-blind acute cross-over design, with cognitive and mood assessments pre-dose, 40-mins and 120-mins post-dose. The primary outcome measure was accuracy in Rapid Visual Information Processing (RVIP). Secondary cognitive outcome measures included RVIP reaction time as well as Inspection time (IT), Jensen Box decision/reaction times, serial subtraction and N-Back working memory. Secondary mood measures included Bond-Lader and caffeine Research visual analogue scales (VAS). No significant treatment effects were found for the primary outcome measure, although significant effects were found amongst secondary measures. Overall, CGA in isolation was not found to significantly improve cognitive function relative to placebo whereas the GB was found to improve sustained attention as measured by the N-Back task in comparison to placebo overall (t=2.45,p=.05), as well as decision time on a 2-choice reaction time task (Jensen box) in comparison to placebo at 40 minutes post-dose (t=2.45,p=.05). Similarly, GB was found to improve alertness on both the Bond-Lader at 120 minutes relative to CGA (t=2.86, p=0.02) and the caffeine Research VAS relative to CGA (t=3.09, p=0.009) and placebo (t=2.75,p=0.02) at 120 minutes post-dose. Both the GB and CGA were also found to significantly improve symptoms of headache at 120 minutes relative to placebo (t=2.51,p=0.03 and t=2.43,p=.04 respectively), whilst there was a trend towards a reduction in jitteriness with GB and CGA in comparison to placebo at 40 minutes post-dose (t=2.24,p=0.06 and t=2.20,p=0.06 respectively). These findings suggest that the improvements in mood observed with GB, but not the improvements in cognitive function, are likely to some extent to be attributable to CGAs.

Trial Registration: Australia New Zealand Clinical Trials Registry ACTRN12611000067976 www.anzctr.org.au

## Introduction

Coffee is one of the most widely consumed beverages throughout the world. The physiological and behavioural effects of coffee (with caffeine) have been extensively studied over the past four decades with research generally demonstrating that caffeine exerts positive effects on cognitive performance, including measures of reaction time (RT), alertness, and vigilance [1-9]. In contrast, little is known regarding the cognitive and mood effects of non-caffeine constituents of coffee, as their effects are difficult to dissociate from those of caffeine. This is due in part to the significant effects on brain function which are exerted by caffeine alone [10,11], but is also due to the effects of non-caffeine compounds in modulating or even opposing those of caffeine [12]. For this reason the acute effects of other compounds found in coffee are difficult to observe, except in decaffeinated coffee.

 Chlorogenic acids (CGA) are polyphenolic compounds which make up 7-9% of coffee by weight, in contrast to caffeine which makes up as little as 1% [13]. CGAs include *caffeoylquinic* acid, *feruloylquinic* acid, *dicaffeoylquinic* acid and smaller quantities of *p*-*coumaroylquinic* acid. CGAs from a variety of plant sources have been shown to exert cardio-protective and anti-oxidant effects as well as the inhibition of lipid peroxidation [14-24]. A recent study by Kwon et al. [25] which used a scopolamine animal model of amnesia, showed that CGAs inhibit acetylcholinesterase activity *in vitro*. Through effects on serotonin transmission, CGAs have also been shown to exert anxiolytic effects in an animal model of restraint stress [26]. 

In a recent exploratory trial from our laboratory [27], it was found that a decaffeinated green coffee blend (NESCAFÉ Green Blend coffee) high in CGAs (521mg) exerted a number of trend-level effects on cognition and mood, including increased alertness and decreased headaches and mental fatigue in comparison to regular decaffeinated coffee. As CGAs are the most abundant family of compounds found in decaffeinated green blend coffee, it is feasible that the positive cognitive and mood effects observed in the study by Cropley et al. [27] are attributable to CGAs. To further explore this possibility, the present study aimed to assess the effect of pure CGA on cognitive function and mood in a healthy older population. To better understand whether it is the CGAs in the decaffeinated green coffee blend that are attributed to the positive effects, a second aim was to compare the effects of pure CGA with the decaffeinated green coffee blend. If it is CGAs contributing to the positive effects, no difference in cognitive performance and mood between pure CGA and decaffeinated green blend coffee is expected. Finally, as the results from the initial acute clinical trial [27] were the first indication that a non-caffeinated pure coffee may improve cognitive performance, the third aim was to replicate and confirm the previous findings and show that six grams of decaffeinated green blend coffee improves attention and mood in a healthy older population. To address these research aims, Rapid Visual Information Processing (RVIP) accuracy was used as the primary outcome, and a range of secondary cognitive and mood measures were also used in order to explore differential treatment effects associated with decaffeinated green blend coffee in comparison to pure CGA.

## Methods

### Participants

**Figure 1:**
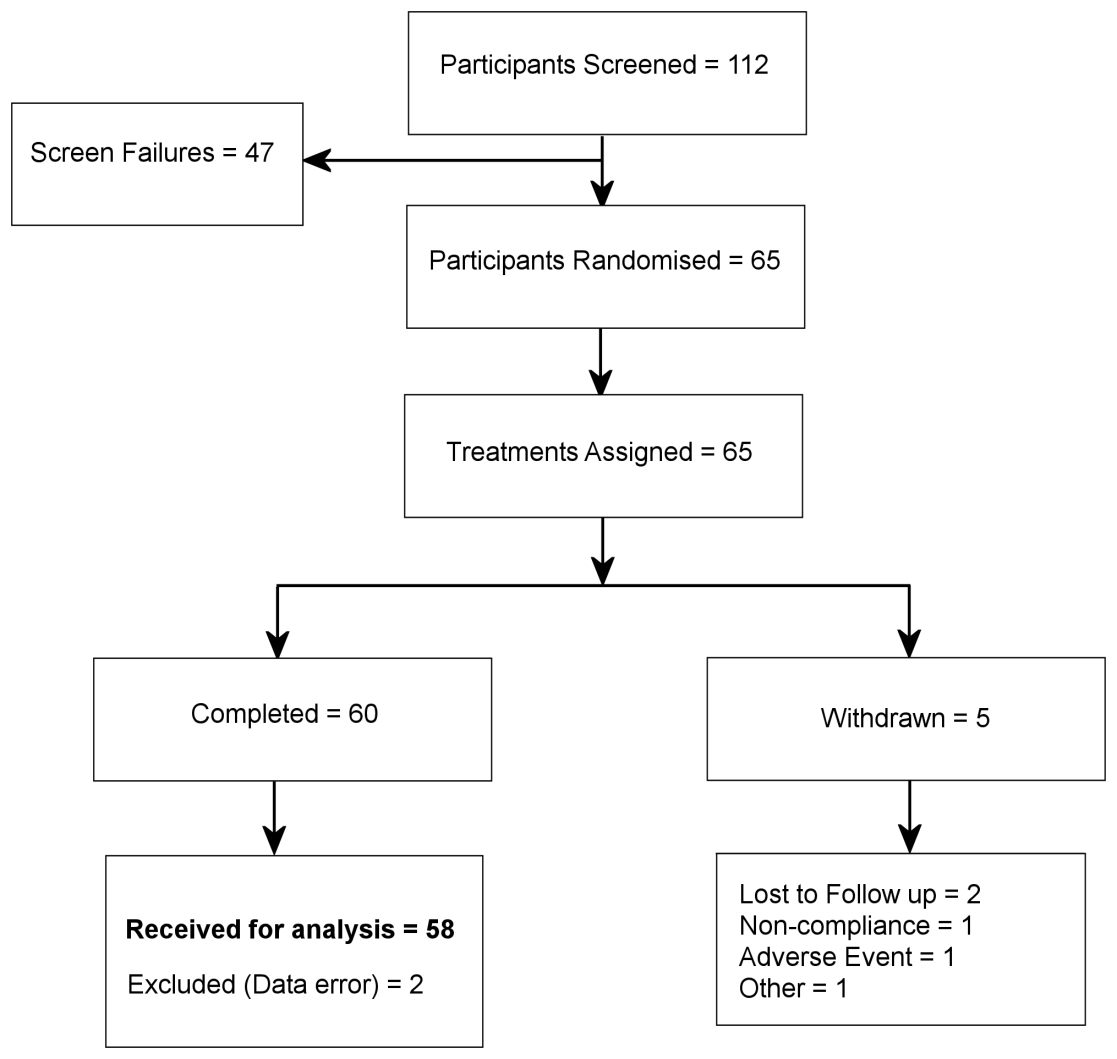
Trial Profile Plot.

112 Participants were initially screened to take part in the study, resulting in 65 patients who met inclusion criteria and were randomized to receive treatments. Out of those randomized, 60 participants completed and were included in the final analysis. Demographic characteristics of participants at baseline are displayed in Table 1 and the trial design is detailed in Figure 1. Sample size was calculated on the basis of power analysis using previous data from our laboratory [27] regarding the primary outcome measure of RVIP accuracy (see below). The estimated effect size for RVIP accuracy was an increased score of 2.45 points for decaffeinated coffee from green beans in comparison to placebo, with a within-subject SD of 6.52 points. Using this effect size, and assuming 80% power of detecting this effect in a repeated-measures design, a sample size of N=58 was calculated according to a 5% (two-tailed) significance level. 

**Table 1:** Participant baseline demographic characteristics.

*Gender*		
	Male	43% (26)
	Female	57% (34)
*Age (years)*		
	Lower quartile	60
	Median	64.5
	Upper quartile	68
*Education*		
	< 4 years	5% (3)
	4 - 8 years	30% (18)
	8-9 years	13% (8)
	> 10 years	52% (31)
*Body Mass Index*		
	Lower quartile	22.8
	Median	24.6
	Upper quartile	27.325

All participants were healthy older adults aged 50+ years, as well as being light to moderate coffee drinkers (i.e. with caffeine) drinking no more than 16 cups of coffee per week. Participants without any existing or pre-existing physical or neurological conditions, no history of psychiatric, cardiac, endocrine, gastrointestinal, or bleeding disorders, not taking any medication that could potentially affect the outcome of the study (i.e. psychoactive medication) including drugs, excessive amounts of alcohol, and non-smokers, were included in the study. An MMSE score of 27 or higher was an additional inclusion criterion, and was taken as evidence of normal cognitive functioning. Participants were also required to abstain from any caffeine-containing or CGA-rich drinks or food products for 24 hours prior to each testing session. All participants provided written informed consent and the study was approved by the Swinburne University Ethics Committee. Australia New Zealand clinical trials registration number: *ACTRN12611000067976*. The protocol for this trial and supporting CONSORT checklist are available as supporting information; see Checklist S1 and Protocol S1.

### Study design and treatments

The study was an acute-dose cross-over, placebo-controlled, double blind, randomized, single centre, clinical trial with three treatment conditions: Chlorogenic acid (CGA), decaffeinated green blend coffee and placebo. The CGA treatment consisted of 540mg chlorogenic acid mixed in a placebo matrix of 5460 mg maltodextrin and a soluble powder derived from rice cereal (CHE; Cereals Hydrolzed Enzymatically). Coffee flavour and colour were added in order to maintain treatment blinding. The placebo treatment consisted of 6g maltodextrin and CHE, also with coffee flavour and colouring. Decaffeinated green blend coffee (NESCAFÉ Green Blend, Nestlé Switzerland), is a soluble coffee manufactured from a blend of decaffeinated green and roasted coffee beans, resulting in an increased CGA content in comparison to regular coffee. 6g of green blend coffee was administered in the current study. For each of the three treatments, the composition of chlorogenic acids and caffeine are listed in Table 2. The CGA and green blend decaffeinated coffee treatments were well-matched for total CGA dosage, whilst the difference in caffeine dosage between treatments was small (3.8mg). All treatments were in powdered form and were dissolved and administered as a normal hot coffee drink. One coffee sachet (6 grams) was dissolved in 300 milliliters of boiled water, with milk and saccharine added by participants to suit their own taste. The addition of milk to black coffee has not been found to affect the bioavailability of decaffeinated green blend coffee [28]. The three treatments were coded as A, B and C and a randomization schedule for each subject was generated using Williams Design in R 2.6.1 by a Nestle Biostatistician not involved in the study.

**Table 2:** Composition of the study coffee treatments per serving (mg/g).

		Green Blend decaffeinated coffee	CGA	Placebo
***Chlorogenic acids (mg/g)***		88.6	93.0	0.00
	3-CQA	20.1	15.2	0.00
	4-CQA	23.5	24.3	0.00
	5-CQA	25.8	25.6	0.00
	3,4di-CQA	3.2	6.0	0.00
	3,5di-CQA	1.8	3.6	0.00
	4,5di-CQA	2.8	5.5	0.00
	4-FQA	4.2	5.4	0.00
	5-FQA	7.2	7.4	0.00
	Caffeic acid	0.1	0.9	0.00
***Caffeine (mg/g)***		0.8	1.6	0.1
***Per Serving***		(6g)	(5.7g)	(6g)
***TotalCGAs*** (***mg/cup***)		532	530	0.0
***TotalCaffeine(mg/cup***)		5.0	8.8	0.7

Total CGAs and Caffeine according to cup.

### Cognitive measures

#### Rapid Visual Information Processing (RVIP)

The RVIP task is a test of sustained attention which loads heavily on working memory. Single digits (1-9) are presented continuously in the middle of a computer screen in a semi-random order. Participants are required to press the response button as soon as they detect three consecutive odd or three even digits in ascending order (i.e. 2,4,6; 3,5,7; 4,6,8; 5,7,9). The digits are presented at a rate of 100 digits per minute. Stimulus duration is 600ms with no inter-stimulus interval. There are eight target sequences (i.e. potential correct hits) presented per minute (96 targets in total). The total duration of the task is 12 minutes. The outcome measures are the total number of correct hits (accuracy), reaction time for correct hits and false alarm rate. RVIP accuracy was used as the primary outcome measure.

#### N Back Task

The N-Back task is a measure of sustained and selective attention and impulsivity. Single digits are presented on the screen. Participants are required to press ‘YES’ or ‘NO’ using a button box, to indicate whether the digit is the same as the *n* previously (e.g. 1-back the previous digit, 3-back the digit 3 previously). The task is scored for speed and accuracy and the task duration is approximately 4 minutes. 

#### Inspection Time (IT)

The IT task is a measure of perceptual speed. This task assesses the presentation time that a subject requires to discriminate between two possible stimuli. The task consists of a stimulus with two vertical parallel lines joined at the top by a horizontal line. There are two versions of the stimulus; either the left line is shorter than the right or the right line is shorter than the left. Stimuli are flashed on a computer screen and the participant is instructed to press a key corresponding to the side of the symbol that is shorter. Each stimulus presentation is followed by the presentation of a backward visual mask. This prevents further processing of the stimulus in iconic memory. The speed of stimulus presentation is varied according to the accuracy of the participants’ responses. The length of presentation of the backward visual mask also varies to determine the optimal visual encoding time. The objective is to respond as accurately, rather than as quickly, as possible. The duration of stimulus presentation is varied until an 80% accuracy level is obtained by the participant. This is taken as the outcome measure for speed of visual information processing speed.

#### Jensen Box

The Jensen Box is an apparatus that distinguishes simple and choice decision time and movement time from total reaction time. This apparatus has eight lights which are arranged in a semi-circular configuration. A response button is located adjacent to each light. A "home" button is situated in the centre of the panel. Participants were required to keep the home button pressed down until a target light appeared. When the target light appeared they were then required to release the home button as quickly as possible and press the response button adjacent to the stimulus light. Decision time (DT) was defined as the time from stimulus onset to the release of the home button, and movement time (MT) as the time from release of the home button to the depression of the stimulus button. Choice was manipulated by varying the number of stimulus alternatives, from 0 (i.e. one light at one possible location) to multiple (i.e. the stimulus may appear in any one of the eight light positions). Participants were given several practice trials in the eight stimulus (i.e. eight lights) condition so that they could familiarise themselves with the task. DTs of less than 150 ms were discarded as outliers, as it has been argued that physiological limits prevent shorter DTs (Jensen, 1987). DTs over 999 ms were also discarded and replaced with an additional trial. In addition, all DTs exceeding three SDs above the subject's mean DT were also discarded (Jensen, 1987). The outcome measures were the mean DTs and MTs for all four choices: 1, 2, 4 and 8. 

#### Serial Threes and Sevens

Serial sevens is a computerised task measuring concentration and working memory. A random starting number between 800 and 999 is presented on the computer screen. Participants are required to count backwards in sevens from the given number, as quickly and as accurately as possible, with responses made using a numeric keyboard. The outcome measures are the total number of subtractions and number of errors. The duration of the task is 2 minutes. Serial Threes is identical to Serial Sevens except that it requires serial subtractions of threes. The duration of the task is also 2 minutes.

### Mood measures

#### Bond-Lader Visual Analogue Mood Scales (B-L VAS)

The B-L scales provide self-evaluation of mood. In total, 16 dimensions of mood are given: Alert-Drowsy, Calm-Excited, Strong-Feeble, Muzzy-Clear headed, Well Coordinated-Clumsy, Lethargic-Energetic, Contented-Discontented, Troubled-Tranquil, Mentally Slow-Quick Witted, Tense-Relaxed, Attentive-Dreamy, Incompetent-Proficient, Happy-Sad, Antagonistic-Friendly, Interested-Bored, Withdrawn-Social. The participant is required to mark, on a 100 mm line to what extent the described state is appropriate to him/her at that moment in time. The individual responses from the 16 mood scales are combined to make three affective dimensions of alertness, contentment, and calmness. 

#### Caffeine Research Visual Analogue Scales (Caff-VA*S*)

The Caff-VAS scales consist of seven visual analogue scales ("relaxed", "alert", "jittery", "tired", "tense", "headache", overall mood") that have previously been used in research into the effects of caffeine (Rogers et al., 2003). In addition, a single "mentally fatigued" visual analogue scale was included, as previous research has shown it to be sensitive to a caffeine-glucose drink (Kennedy & Scholey, 2004). 

### Statistical analysis

 All statistical analyses were conducted using SAS 9.2 with linear mixed modelling (PROC MIXED). The multivariate correlated error approach was used, whereby within-subject parameter estimates of residual covariance were estimated for each combination of time and treatment. The Residual Maximum Likelihood estimation method (REML) was used with unstructured (UN) covariance and a between-within degrees of freedom method. Fixed terms were fitted for the Treatment Effect (Placebo, Green Blend, CGA) and Time since dosing (40 mins, 120 mins). Pre-dose performance was fitted as a covariate and the interaction of Treatment*Visit was specified as the repeated term. Post-hoc differences of least squares means were used to determine significant differences between treatment groups overall, as well as at individual time points (40 min and 120 min). At each timepoint (40 min and 120 min) significance values for least square means comparing the three treatment groups were adjusted for multiplicity using the Hommel method. An adjusted alpha level of 0.05 was set for statistical significance of least square differences, however to allow greater exploration of the data any least square differences associated with corrected p-values <.10 were taken as trend level and have also been discussed in text. Cohen’s d effect sizes were calculated for significant differences of least square means. 

### Procedure

A short telephone interview was conducted with all potential subjects to ensure study criteria were met. Successful volunteers attended one training session. Participants were asked to abstain from alcohol, caffeinated and CGA-rich foods/beverages, and to consume foods with low polyphenol content for 24 hours prior to each testing session. During the training session participants completed demographic and medical questionnaires, the Mini Mental State Examination (MMSE), and signed informed consent forms (if criteria met). Following this, participants completed the battery of cognitive tests to minimize procedural learning effects and familiarize subjects with study measures. Participants attended four study visits separated by a one week washout period. The first visit was a training session (V0), and the remaining three experimental sessions (V1, V2, V3) participants were randomized to receive CGA, decaffeinated Green Blend or placebo. Cognitive and mood assessments were conducted at three time points for each study visit: before treatment (pre-dose), 40 minutes and 120 minutes after treatment (post-dose). Individual assignment to the order of treatment condition was randomized and counterbalanced according to stratification per gender. This was provided as a list by the Nestlé Research Centre (NRC) biostatistician.

At the beginning of each experimental session, a sandwich (containing salad with either cheese or chicken) and water were provided. After the participant finished eating, subjects completed the mood visual analogue scales followed by the cognitive tests. The mood scales were completed a second time immediately after baseline cognitive testing. The battery of tests were approximately 40 minutes’ duration. Subjects were then administered the equivalent of three servings of coffee, consumed within 15 minutes. Based on a previous pharmacokinetic study conducted at Nestlé, following oral ingestion of decaffeinated NESCAFÉ Green Blend soluble coffee, several polyphenol metabolites were shown to peak in plasma within two hours after coffee ingestion [29]. Based on these results and the results from the previous pilot decaffeinated coffee trial from our laboratory [27], cognitive and mood testing began 40 minutes after treatment consumption. Participants rested for approximately 15 minutes before the final battery of cognitive and mood tests were administered 120 minutes following treatment consumption. All data was collected at Swinburne University of Technology, Hawthorn, Australia between November 2010 and July 2011. Each experimental session took approximately four hours to complete and was separated by a minimum one week washout period. 

## Results

### Cognitive outcome measures

**Figure 2:**
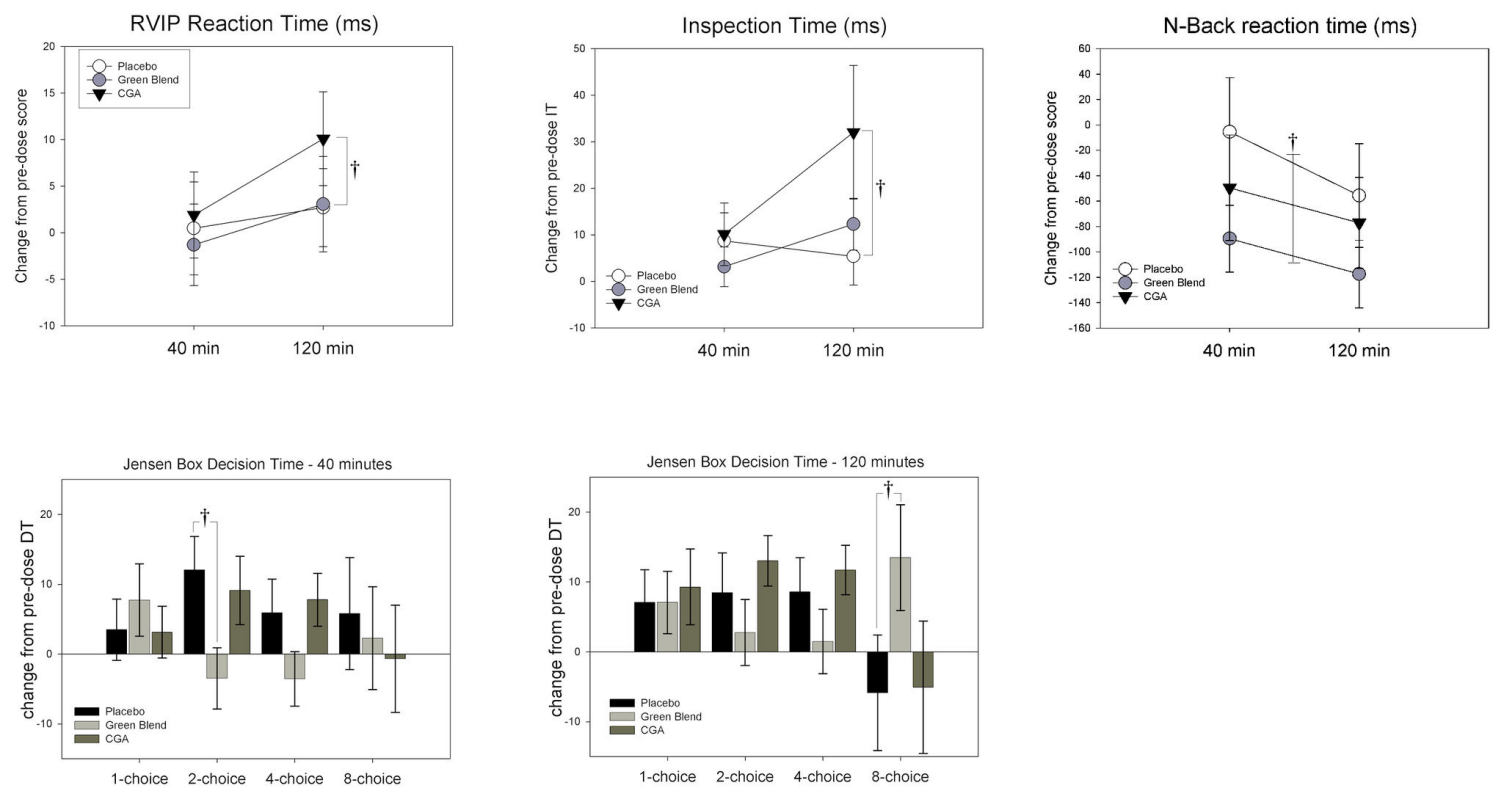
Significant Treatment effects on Cognitive Measures. † p<.10, * p<.05, p<.01.

Means and standard errors of responses on all cognitive outcome measures are displayed in Table 3 according to treatment group. Significant differences in cognitive outcome measures between treatment groups at 40 minute and 120 minute time points are displayed in Figure 2.

**Table 3:** Means (±SE) of cognitive outcome measures.

	Treatment	*N*	*pre-dose*	*40 mins*	*120 mins*
RVIP -Accuracy (%)	*Placebo*	58	44.94 (2.64)	44.04 (2.65)	43.28 (2.87)
	*Green Blend*	58	45.03 (2.72)	45.46 (2.76)	44.79 (2.87)
	*CGA*	58	46.52 (2.79)	44.54 (2.91)	44.49 (2.83)
RVIP -RT (ms)	*Placebo*	58	541.8 (7.63)	542.3 (7.64)	544.5 (7.99)
	*Green Blend*	58	548.3 (7.32)	547.0 (7.26)	551.3 (7.95)
	*CGA*	58	547.5 (7.53)	549.4 (7.48)	557.6 (8.08)
RVIP -False Alarms	*Placebo*	58	19.43 (4.23)	21.79 (4.63)	19.43 (4.30)
	*Green Blend*	58	17.60 (2.83)	24.50 (5.23)	23.00 (5.30)
	*CGA*	58	18.55 (2.90)	23.90 (5.85)	16.53 (2.93)
Inspection Time (ms)	*Placebo*	60	101.5 (9.35)	110.2 (10.77)	106.8 (11.13)
	*Green Blend*	60	92.92 (5.14)	96.04 (4.72)	105.2 (7.37)
	*CGA*	60	89.00 (5.08)	99.13 (7.04)	121.0 (15.41)
Jensen 1-choice -DT	*Placebo*	60	331.9 (7.89)	335.4 (6.86)	339.0 (8.16)
	*Green Blend*	60	332.7 (7.75)	340.4 (8.35)	339.7 (7.65)
	*CGA*	60	331.4 (7.09)	334.5 (7.55)	340.6 (8.42)
Jensen 1-choice -MT	*Placebo*	60	620.1 (14.74)	629.2 (14.48)	629.3 (15.43)
	*Green Blend*	60	616.8 (13.64)	635.6 (16.10)	635.6 15.45)
	*CGA*	60	615.0 (13.20)	626.7 (14.83)	636.6 (16.80)
Jensen 2-choice -DT	*Placebo*	60	342.9 (7.49)	354.9 (6.99)	351.3 (7.51)
	*Green Blend*	60	354.2 (8.33)	350.7 (7.70)	356.9 (7.53)
	*CGA*	60	344.4 (6.84)	353.6 (8.07)	357.5 (6.95)
Jensen 2-choice -MT	*Placebo*	60	641.0 (13.80)	662.3 (14.32)	659.2 (14.32)
	*Green Blend*	60	646.5 (14.32)	655.8 (14.32)	665.6 (15.06)
	*CGA*	60	638.0 (13.55)	653.4 (14.92)	667.3 (14.94)
Jensen 4-choice -DT	*Placebo*	60	363.5 (7.76)	369.4 (7.67)	372.1 (8.27)
	*Green Blend*	60	368.9 (8.04)	365.4 (7.35)	370.4 (7.10)
	*CGA*	60	362.2 (7.14)	370.0 (7.09)	373.9 (7.21)
Jensen 4-choice -MT	*Placebo*	60	671.0 (14.83)	678.2 (14.16)	684.6 (15.01)
	*Green Blend*	60	678.3 (14.59)	678.3 (14.44)	683.7 (14.55)
	*CGA*	60	665.7 (14.18)	678.9 (13.96)	689.8 (14.73)
Jensen 8-choice -DT	*Placebo*	60	422.1 (10.46)	427.9 (9.03)	416.2 (7.89)
	*Green Blend*	60	421.8 (10.12)	424.1 (10.79)	435.2 (10.13)
	*CGA*	60	429.4 (12.15)	428.7 (9.68)	424.3 (9.01)
Jensen 8-choice -MT	*Placebo*	60	757.5 (16.85)	764.1 (15.86)	747.4 (14.44)
	*Green Blend*	60	758.6 (16.40)	758.7 (15.45)	766.2 (17.29)
	*CGA*	60	759.0 (19.85)	765.7 (17.40)	757.8 (16.01)
Serial 3s -Correct	*Placebo*	60	26.67 (1.42)	27.48 (1.34)	27.88 (1.37)
	*Green Blend*	60	26.51 (1.62)	27.59 (1.54)	28.25 (1.52)
	*CGA*	60	26.97 (1.40)	27.65 (1.46)	28.42 (1.56)
Serial 3s -RT (ms)	*Placebo*	60	4782 (268.2)	4404 (196.7)	4415 (227.2)
	*Green Blend*	60	4828 (289.0)	4457 (227.0)	4572 (253.3)
	*CGA*	60	4732 (259.5)	4436 (228.1)	4370 (222.6)
Serial 7s -Correct	*Placebo*	60	18.98 (1.13)	18.40 (1.16)	19.13 (1.15)
	*Green Blend*	60	18.90 (1.16)	18.68 (1.22)	19.31 (1.24)
	*CGA*	60	18.33 (1.18)	18.97 (1.20)	19.66 (1.12)
Serial 7s -RT (ms)	*Placebo*	60	6597 (396.4)	6554 (375.5)	6563 (408.7)
	*Green Blend*	60	6663 (404.3)	6875 (528.4)	6493 (404.0)
	*CGA*	60	6671 (458.1)	6628 (430.6)	6313 (403.8)
N-Back -Accuracy (%)	*Placebo*	60	83.22 (1.14)	83.26 (1.22)	83.44 (1.16)
	*Green Blend*	60	81.67 (1.26)	83.82 (1.01)	83.22 (1.21)
	*CGA*	60	81.59 (1.21)	82.41 (1.11)	84.52 (1.12)
N-Back -RT (ms)	*Placebo*	60	1142 (52.84)	1137 (57.55)	1086 (52.48)
	*Green Blend*	60	1131 (53.98)	1041 (46.93)	1013 (47.35)
	*CGA*	60	1125 (52.75)	1076 (58.43)	1048 (45.78)

#### Rapid Visual Information Processing (RVIP)

Two participants were removed from analysis due to zero percent accuracy on at least one of the time points. Mixed ANCOVA adjusting for baseline scores and considering time, treatment and their interaction as independent variables were fitted for accuracy, reaction time (RT) and number of false alarms. No significant treatment effect or interaction with time was found for the primary outcome measure RVIP accuracy, or for RVIP reaction time. However, differences of least square means revealed that at 120 minutes post-dose there was a trend towards RT being slower with CGA consumption in comparison to placebo (t=-2.25, p=.08, d=0.42).

#### Inspection (IT)

Box-cox transformation was applied to IT responses (Lambda = -0.5) in order to normalize the data prior to analysis. Mixed ANCOVA adjusting for baseline IT and considering time, treatment and their interaction as independent variables were fitted to responses. No significant treatment effect or interaction with time was found, although differences of least square means revealed that at 120 minutes post-dose IT was significantly slower with CGA consumption in comparison to placebo (t=-2.26, p=.08, d=0.41).

#### Jensen Task - Decision Time (DT)

Box-cox transformation was applied to all DT responses (Lambda=0, LN) in order to normalize the data prior to analysis. Mixed ANCOVA adjusting for baseline scores and considering time, treatment and their interaction as independent variables were fitted for 1-choice, 2-choice, 4-choice and 8-choice DT. For 2-choice DT there was a trend towards an interaction between treatment and time (F(2,59)=2.36,p=0.10), with differences of least square means revealing a trend towards DT being faster with decaffeinated green blend coffee in comparison to placebo at 40 minutes (t=2.45,p=.05, d=0.45). For 4-choice DT there was a trend towards a main effect for treatment F(2,59)=2.58,p=.08), although no significant between group differences were found with least squares means. For 8-choice DT there was a significant Treatment x time interaction (F(2,59)=3.78,p=0.03), with differences of least square means revealing a trend towards DT being slower with green blend coffee in comparison to placebo at 120 minutes (t=-2.27,p=.08, d=0.41).

#### Jensen Task - Movement Time (MT)

Box-cox transformation was applied to all MT responses (Lambda = 0, LN) in order to normalize the data prior to analysis. Mixed ANCOVA adjusting for baseline scores and considering time, treatment and their interaction as independent variables were fitted for 1-choice, 2-choice, 4-choice and 8-choice MT. For 2-choice MT there was a trend towards an interaction between treatment and time (F(2,59)=2.74,p=0.07), although no significant between group differences were found with least squares means. For 4-choice MT there was a trend towards a main effect for treatment (F(2,59)=2.58,p=0.08), although no significant between groups differences were found with least squares means.

#### Serial 3s and 7s subtraction

Box-cox transformation was applied to RTs (Lambda = -0.5) as well as Total Correct (Lambda = +0.5) in order to normalize the data prior to analysis. Mixed ANCOVA adjusting for baseline scores and considering time, treatment and their interaction as independent variables were fitted for RTs and total numbers correct. No significant treatment effect or interaction with time was found for RTs or total correct in either Serial 3s or Serial 7s. 

#### N-Back

Box-cox transformation was applied to RT (Lambda = 0, LN) in order to normalize the data prior to analysis. Mixed ANCOVA adjusting for baseline scores and considering time, treatment and their interaction as independent variables were fitted for RTs and Accuracy. A significant main effect for treatment was found F(2,59)=3.12,p=.05), with differences of least square means revealing faster RT with Green Blend in comparison to placebo (averaged across both time points) (t=2.45,p=.05, d=0.45). For N-Back accuracy, a trend towards an interaction between treatment and time was found (F(2,59)=2.71,p=.07), although no significant between group differences were found with least square means. 

### Mood outcome measures

**Figure 3:**
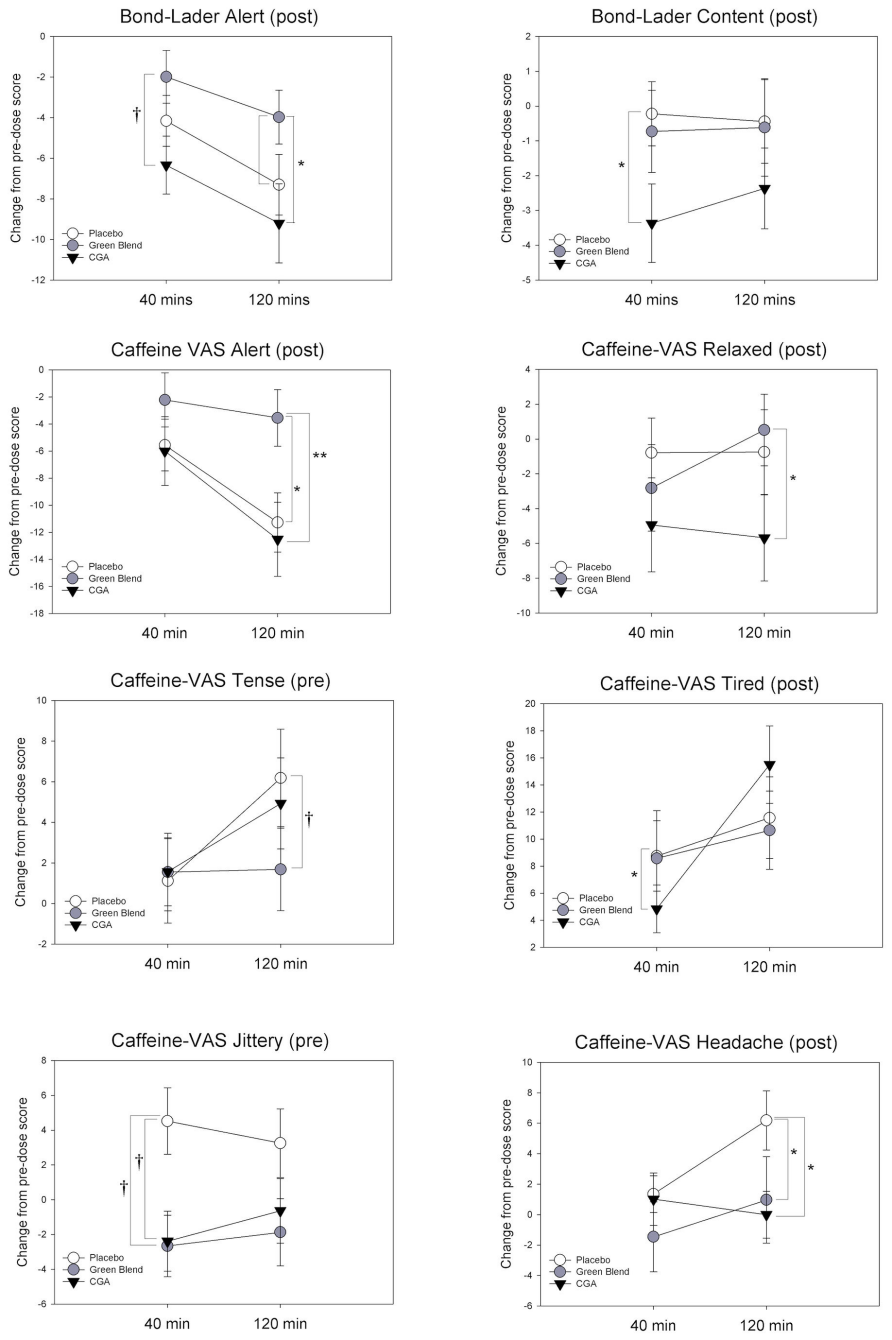
Significant Treatment effects on Mood Measures. † p<.10, * p<.05, p<.01.

Means and standard errors of responses on all mood outcome measures are displayed in Table 4 according to treatment group. Significant differences in mood outcome measures between treatment groups at 40 minute and 120 minute time points are displayed in Figure 3. 

**Table 4:** Means (±SE) of mood outcome measures.

	Treatment	*N*	*pre-dose*	*40 mins*	*120 mins*
Bond-Lader Alert	*Placebo*	60	69.61 (2.04)	71.08(2.18)	67.73 (2.06)
(PRE)	*Green Blend*	60	69.20 (2.07)	72.78 (2.01)	68.30 (2.41)
	*CGA*	60	65.69 (2.22)	71.00 (2.20)	68.39 (2.30)
Bond-Lader Alert	*Placebo*	60	64.06 (2.48)	59.90 (2.75)	56.76 (2.64)
(POST)	*Green Blend*	60	63.48 (2.53)	61.49 (2.60)	59.51 (2.72)
	*CGA*	60	66.29 (2.35)	59.95 (2.59)	57.09 (2.61)
Bond-Lader Content	*Placebo*	60	80.06 (1.52)	79.25 (1.73)	76.76 (2.00)
(PRE)	*Green Blend*	60	78.20 (1.76)	77.79 (1.81)	76.69 (1.99)
	*CGA*	60	78.93 (1.58)	77.78 (1.87)	77.85 (1.87)
Bond-Lader Content	*Placebo*	60	73.22 (2.16)	73.00 (2.26)	72.78 (2.27)
(POST)	*Green Blend*	60	73.08 (2.20)	72.35 (2.27)	72.46 (2.33)
	*CGA*	60	74.58 (2.07)	71.22 (2.36)	72.22 (2.32)
Bond-Lader Calm	*Placebo*	60	75.29 (1.87)	73.84 (2.09)	71.66 (2.20)
(PRE)	*Green Blend*	60	75.07 (1.84)	73.37 (1.98)	72.20 (2.18)
	*CGA*	60	74.88 (1.85)	74.93 (2.02)	72.59 (2.07)
Bond-Lader Calm	*Placebo*	60	66.90 (2.41)	67.23 (2.59)	67.88 (2.42)
(POST)	*Green Blend*	60	67.70 (2.48)	67.30 (2.23)	66.75 (2.65)
	*CGA*	60	68.16 (2.56)	67.40 (2.27)	67.10 (2.50)
CaffVAS Alertness	*Placebo*	60	70.70 (2.25)	68.67 (2.35)	63.07 (2.37)
(PRE)	*Green Blend*	60	69.30 (2.49)	69.88 (2.37)	65.08 (2.81)
	*CGA*	60	68.57 (2.67)	68.25 (2.47)	62.10 (2.92)
CaffVAS Alertness	*Placebo*	60	62.67 (2.76)	57.12 (2.74)	51.40 (2.84)
(POST)	*Green Blend*	60	60.20 (2.95)	58.42 (2.95)	56.65 (2.81)
	*CGA*	60	61.95 (2.78)	55.95 (3.11)	49.43 (2.80)
CaffVAS Relaxation	*Placebo*	60	71.42 (2.58)	72.95 (2.17)	70.30 (2.19)
(PRE)	*Green Blend*	60	72.62 (2.47)	70.55 (2.42)	68.07 (2.63)
	*CGA*	60	73.05 (2.33)	71.65 (2.44)	67.33 (2.62)
CaffVAS Relaxation	*Placebo*	60	64.02 (2.77)	63.23 (2.95)	63.27 (2.64)
(POST)	*Green Blend*	60	65.22 (2.75)	62.27 (2.91)	65.73 (2.87)
	*CGA*	60	65.33 (2.68)	60.40 (3.25)	59.65 (2.96)
CaffVAS Mental Fatigue	*Placebo*	60	21.65 (2.21)	31.72 (2.79)	37.08 (2.69)
(PRE)	*Green Blend*	60	21.73 (2.48)	32.62 (3.03)	37.42 (3.18)
	*CGA*	60	22.75 (2.67)	29.73 (2.76)	37.77 (3.33)
CaffVAS Mental Fatigue	*Placebo*	60	37.58 (3.11)	44.77 (3.11)	52.02 (3.23)
(POST)	*Green Blend*	60	38.22 (3.14)	45.31 (3.30)	48.67 (3.29)
	*CGA*	60	37.22 (3.06)	45.62 (3.10)	53.55 (3.10)
CaffVAS Tiredness	*Placebo*	60	26.50 (2.27)	34.03 (2.84)	40.45 (2.62)
(PRE)	*Green Blend*	60	26.75 (2.60)	32.92 (2.71)	36.25 (2.67)
	*CGA*	60	29.62 (2.93)	33.33 (2.74)	36.97 (2.69)
CaffVAS Tiredness	*Placebo*	60	38.38 (2.89)	47.13 (3.02)	49.95 (2.83)
(POST)	*Green Blend*	60	35.98 (2.81)	44.39 (3.23)	46.63 (3.00)
	*CGA*	60	34.95 (2.82)	39.80 (2.76)	50.45 (2.81)
CaffVAS Jittery	*Placebo*	60	14.45 (1.57)	18.97 (2.24)	17.70 (2.14)
(PRE)	*Green Blend*	60	18.97 (2.18)	16.32 (1.79)	17.10 (2.06)
	*CGA*	60	18.73 (2.26)	16.35 (1.94)	18.10 (2.12)
CaffVAS Jittery	*Placebo*	60	22.15 (2.62)	21.90 (2.50)	18.33 (2.15)
(POST)	*Green Blend*	60	20.37 (2.38)	19.76 (2.49)	20.47 (2.55)
	*CGA*	60	20.85 (2.44)	19.30 (2.33)	21.17 (2.68)
CaffVAS Headache	*Placebo*	60	13.07 (2.09)	15.58 (2.44)	19.00 (2.69)
(PRE)	*Green Blend*	60	16.23 (2.63)	16.02 (2.74)	20.87 (3.27)
	*CGA*	60	13.65 (2.06)	15.82 (2.39)	17.42 (2.70)
CaffVAS Headache	*Placebo*	60	15.03 (2.34)	16.38 (2.45)	21.22 (2.98)
(POST)	*Green Blend*	60	18.98 (3.09)	17.68 (2.98)	19.95 (3.35)
	*CGA*	60	17.18 (2.32)	18.20 (2.61)	17.18 (2.49)
CaffVAS Tension	*Placebo*	60	19.85 (1.98)	20.97 (2.12)	26.03 (2.41)
(PRE)	*Green Blend*	60	20.78 (2.14)	22.33 (2.46)	22.47 (2.36)
	*CGA*	60	19.85 (2.04)	21.42 (2.10)	24.78 (2.47)
CaffVAS Tension	*Placebo*	60	29.85 (2.79)	29.37 (2.84)	31.27 (3.00)
(POST)	*Green Blend*	60	27.23 (2.54)	28.44 (3.06)	30.82 (3.26)
	*CGA*	60	30.88 (2.64)	29.15 (2.62)	30.38 (2.77)
CaffVAS Overall Mood	*Placebo*	60	82.10 (1.84)	77.27 (2.26)	77.05 (2.04)
(PRE)	*Green Blend*	60	79.42 (1.87)	76.53 (2.47)	74.93 (2.40)
	*CGA*	60	79.35 (2.36)	76.37 (2.58)	75.80 (2.48)
CaffVAS Overall Mood	*Placebo*	60	73.12 (2.76)	73.20 (2.54)	72.62 (2.63)
(POST)	*Green Blend*	60	74.35 (2.32)	71.97 (2.74)	69.28 (2.96)
	*CGA*	60	74.97 (2.46)	72.78 (2.45)	71.68 (2.56)

#### Bond-Lader Alert

ANCOVA adjusting for baseline scores and considering time, treatment and their interaction as independent variables were fitted for Bond-Lader Alert scores both before cognitive testing (pre-test) and after cognitive testing (post-test). For pre-test Alert scores no significant treatment effect or interaction with time was found. However, for post-test Alert scores a significant treatment effect was revealed (F(2,59)=4.18,p=.02). Differences of Least Square Means revealed that Alertness was significantly greater with decaffeinated green blendcoffee in comparison to CGA overall (t=2.89, p=.02, d=0.53). At 40 minutes there was a trend towards Alertness being greater with decaffeinated green blend coffee in comparison to CGA (t=2.41, p=.06, d=0.44). At 120 minutes Alertness was found to be significantly greater with the green blend coffee in comparison to CGA (t=2.86, p=.02, d=0.52) and there was also a trend towards Alertness being greater with the green blend coffee in comparison to placebo (t=-2.16, p=.07, d=0.39).

#### Bond-Lader Content

Box-cox transformation was applied to pre-test content scores (Lambda = +2) as well as post-test content scores (Lambda = +1.5) in order to normalize the data prior to analysis. ANCOVA adjusting for baseline scores and considering time, treatment and their interaction as independent variables were fitted for Bond-Lader Content scores both before cognitive testing (pre-test) and after cognitive testing (post-test). For pre-test Content scores no significant treatment effect or interaction with time was found. However, for post-test Content scores a trend towards a main effect for treatment was found (F(2,59)=2.85,p=.07). Differences of Least Square Means revealed a trend towards contentedness being greater with placebo in comparison to CGA overall (t=2.33, p=.07, d=0.42). At 40 minutes Contentedness was found to be significantly greater with Placebo in comparison to placebo (t=2.86, p=.02, d=0.52).

#### Bond-Lader Calm

Box-cox transformation was applied to pre-test calmness scores (Lambda = +1.5) in order to normalize the data prior to analysis. ANCOVA adjusting for baseline scores and considering time, treatment and their interaction as independent variables were fitted for Bond-Lader Calm scores both before cognitive testing (pre-test) and after cognitive testing (post-test). No significant treatment effects or interactions with time were either pre or post-test. 

#### Caffeine-VAS Alert

ANCOVA adjusting for baseline scores and considering time, treatment and their interaction as independent variables were fitted for Caff-VAS Alert scores both before cognitive testing (pre-test) and after cognitive testing (post-test). Pre-test no significant treatment effects or interaction with time was found. However, post-test a significant main effect for Treatment was revealed (F(2,59)=3.40,p=.04). Differences of Least Square Means revealed that there was a trend towards Alertness being greater with decaffeinated green blend coffee in comparison to CGA (t=2.41,p=.05, d=0.44) and placebo (t=2.21,p=.06, d=0.40) overall. At 120 minutes Alertness was found to be significantly greater with the green blend coffee in comparison to placebo (t=2.75, p=.02, d=0.50) and with green blend coffee in comparison to CGA (t=3.09, p=.009, d=0.56). 

#### Caffeine-VAS Relaxed

Box-cox transformation was applied to pre-test relaxation scores (Lambda = +2) in order to normalize the data prior to analysis. ANCOVA adjusting for baseline scores and considering time, treatment and their interaction as independent variables were fitted for Caff-VAS Relaxed scores both before cognitive testing (pre-test) and after cognitive testing (post-test). No significant treatment effects or interaction with time were found either pre or post-test. However differences of Least Square Means revealed that at 120 minutes Relaxation was significantly greater with decaffeinated green blend coffee in comparison to CGA (t=2.75,p=.02, d=0.50). 

#### Caffeine-VAS Tired

Box-cox transformation was applied to pre-test Tiredness scores (Lambda = +0.5) in order to normalize the data prior to analysis. ANCOVA adjusting for baseline scores and considering time, treatment and their interaction as independent variables were fitted for Caff-VAS Tired scores both before cognitive testing (pre-test) and after cognitive testing (post-test). Pre-test no significant treatment effect or interaction with time was found. However, post-test a significant interaction between treatment and time was found (F(2,59)=3.79,p=.03). Differences of Least Square Means revealed that at 40 minutes Tiredness was significantly lower with CGA in comparison to placebo (t=2.53,p=.04, d=0.46). 

#### Caffeine-VAS Mental Fatigue

Box-cox transformation was applied to pre-test headache scores (Lambda = +0.5) in order to normalize the data prior to analysis. ANCOVA adjusting for baseline scores and considering time, treatment and their interaction as independent variables were fitted for Caff-VAS Mental Fatigue scores both before cognitive testing (pre-test) and after cognitive testing (post-test). No significant treatment effect or treatment by time interaction was found either pre or post-test. 

#### Caffeine-VAS Tense

Box-cox transformation was applied to pre-test tension scores (Lambda = +0.25) as well as post-test tension scores (Lambda = +0.5) in order to normalize the data prior to analysis. ANCOVA adjusting for baseline scores and considering time, treatment and their interaction as independent variables were fitted for Caff-VAS Tense scores both before cognitive testing (pre-test) and after cognitive testing (post-test). Pre-test there was a trend towards an interaction between treatment and time (F(2,59)=3.03,p=.06). Differences of Least Square Means revealed that at 120 minutes there was a trend towards Caff-VAS Tense scores being lower with decaffeinated green blend coffee in comparison to placebo (t=2.36,p=.07, d=0.43). Post-test no significant treatment effect or treatment by time interaction was found.

#### Caffeine-VAS Jittery

Box-cox transformation was applied to pre-test Jitteryness scores (Lambda = +0.25) as well as post-test content scores (Lambda = +0.25) in order to normalize the data prior to analysis. ANCOVA adjusting for baseline scores and considering time, treatment and their interaction as independent variables were fitted for Caff-VAS Jittery scores both before cognitive testing (pre-test) and after cognitive testing (post-test). Pre-test there was a trend towards a main effect for Treatment (F(2,59)=2.46,p=.09). Differences of Least Square means revealed a trend towards decreased Jitteriness with decaffeinated green blend coffee in comparison to placebo at 40 minutes (t=2.24,p=.06, d=0.41) and with CGA in comparison to placebo at 40 minutes (t=2.20,p=.06, d=0.40). Post-test there was a trend towards an interaction between treatment and time (F(2,59)=3.13,p=.05), however no significant between-group differences were found with least square means. 

#### Caffeine-VAS Headache

Box-cox transformation was applied to pre-test headache scores (Lambda = 0, LN) as well as post-test content scores (Lambda = 0, LN) in order to normalize the data prior to analysis. ANCOVA adjusting for baseline scores and considering time, treatment and their interaction as independent variables were fitted for Caff-VAS Headache scores both before cognitive testing (pre-test) and after cognitive testing (post-test). Pre-test no significant treatment effect or interaction with time was found. However, post-test a significant interaction between treatment and time was found (F(2,59)=5.93,p=.005). Differences of Least Square Means revealed that at 120 minutes Caff-VAS Headache scores were significantly lower with decaffeinated green blend coffee in comparison to placebo (t=2.51, p=.03, d=0.46) and with CGA in comparison to placebo (t=2.43, p=.04, d=0.44). 

#### Caffeine-VAS Overall Mood Score

Box-cox transformation was applied to pre-test headache scores (Lambda = +2.5) as well as post-test content scores (Lambda = +2) in order to normalize the data prior to analysis. ANCOVA adjusting for baseline scores and considering time, treatment and their interaction as independent variables were fitted for Caff-VAS Mood scores both before cognitive testing (pre-test) and after cognitive testing (post-test). No significant main effects or treatment by time interactions were either pre or post-test. 

## Discussion

### Chlorogenic acids

No significant effects on cognition due to CGA were observed at either 40 minutes or 120 minutes post-dose when compared to placebo. However, there was a trend for CGA to have some negative effects on attention and the speed that information was processed. Specifically, for the RVIP task; whilst no significant effects were found for the primary outcome measure RVIP accuracy, at 120 minutes there was a trend towards CGA consumption being associated with slower RVIP reaction time in comparison to placebo. Similarly, for the Inspection time task at 120 minutes there was a trend towards CGA being associated with longer Inspection time (slower information processing speed) in comparison to Placebo. No other cognitive effects were observed. 

There were few significant CGA mood effects when compared to placebo. Specifically, at 40 minutes (post- cognitive testing), Bond-Lader Content ratings were significantly lower with CGA compared to Placebo (This was corroborated with an overall trend towards lower Content ratings following CGA compared to placebo, irrespective of time). In relation to the Caffeine-VAS questionnaire, at 120 minutes (post- cognitive testing), ratings of Headache symptoms were significantly lower following CGA consumption in comparison to placebo. No other significant mood effects were observed. However, there was a trend towards participants being less jittery following CGA consumption, irrespective of time and at 40 minutes (pre- cognitive testing) specifically. Finally, for the Caffeine-VAS Tiredness scale (prior to cognitive testing), there was a trend towards a decrease in reports of Tiredness at 40-minutes following consumption of CGA in comparison to placebo. In summary, compared to placebo, CGA tended to improve symptoms of tiredness, headaches and jitteriness, yet reduce ratings of contentedness. 

### Effects of decaffeinated green blend coffee

No significant effects of decaffeinated NESCAFÉ Green Blend coffee were found for the primary outcome measure RVIP accuracy. However, compared to placebo decaffeinated green blend coffee significantly improved reaction time on the N-Back working memory task, which was corroborated with a trend towards faster reaction time at both 40 minutes and 120 minutes post-dose. Similarly, for the Jensen-Box 2-choice task, decaffeinated Green Blend coffee significantly improved Decision Time at 40-minutes post-dose in comparison to placebo. Interestingly, in the Jensen-Box 8-choice task, at 120 minutes there was a trend towards slower Decision Time following consumption of decaffeinated green blend coffee in comparison to placebo. No significant treatment effects were found for serial subtraction with threes or sevens, or for the Inspection Time task, suggesting that perhaps only specific cognitive processes are enhanced by decaffeinated green blend. It is also feasible that only the N-Back and the Jensen Box tasks were sensitive enough to detect the subtle changes in cognitive function associated with green blend coffee consumption. In consideration of the fact that the Jensen task is a highly sensitive measure of choice decision time (a component of reaction time), it raises the possibility that lower level cognitive aspects are acutely enhanced more than higher-order cognitive functions with decaffeinated green blend coffee consumption. However, this theory is difficult to reconcile with the fact that improvements were also seen on the N-Back continuous performance task, a task which subsumes both storage and retrieval memory processes in addition to reaction time (i.e. involves a range of cognitive processes including higher order processing). 

The most robust mood effect found to be associated with decaffeinated green blend coffee consumption across both the Bond-Lader and the Caffeine-VAS scales was of acute increases in Alertness. Specifically, decaffeinated green blend coffee improved Bond-Lader Alertness (trend level) at 40 and 120 minutes compared to placebo. This was supported with a significant increase in alertness on the Caffeine-VAS at 120 minutes. It is interesting to note that this effect only emerged after cognitive testing, rather than prior to testing, suggesting that there may be an anti-fatigue effect associated with decaffeinated green blend coffee which helps to maintain Alertness under conditions of mental demand. These improvements in subjective Alertness are in line with the cognitive findings which indicate enhancement of choice reaction time and sustained attention following decaffeinated green blend coffee consumption. These findings also corroborate the findings of our previous pilot study [27] whereby green blend coffee was found to increase alertness in comparison to regular decaffeinated coffee.

Also in line with the previous pilot study by Cropley et al. [27], decaffeinated green blend coffee was found to significantly decrease Caffeine-VAS Headache symptoms at 120 minutes. This effect was corroborated with an overall trend-level reduction in reports of headache symptoms (post- cognitive testing), when compared to placebo, irrespective of time point. In addition, jitteriness, as measured with the Caffeine-VAS, was found to be reduced (at trend level) with decaffeinated green blend coffee compared to placebo (pre- cognitive testing) at 40 minutes specifically and across both time points combined. For the Caffeine-VAS Tiredness scale (pre- cognitive testing), there was also a trend towards a decrease in reports of Tiredness at 120-minutes following consumption of green blend coffee in comparison to placebo. Similarly, for Caffeine-VAS Tension, at 120 minutes (pre- cognitive testing) there was a trend towards a decrease in reports of Tension ratings with decaffeinated green blend coffee compared to placebo. The finding of reductions in Jitteriness and Headache symptoms following decaffeinated green blend coffee consumption are intriguing. In consideration of the fact that these symptoms are typically associated with caffeine withdrawal it raises the possibility that decaffeinated green blend coffee may help to ameliorate withdrawal symptoms and that possibly the amelioration of caffeine withdrawal may also assist in improving select indices of cognition. 

 It is also possible that the very low doses of caffeine present in both the green blend coffee and the CGA treatments (5 mg and 8.8mg respectively) may have had minor effects on amelioration of caffeine withdrawal symtoms. Whilst previous research by Smit and Rogers [3] has suggested that doses as low as 12.5mg caffeine may have effects on cognition and mood following overnight caffeine abstinence, it is important to note that these effects were only reported for heavy habitual caffeine consumers. In consideration of the fact that low-moderate caffeine consumers were included in the current study, it is unlikely that levels of caffeine as low as 5mg would have had a noticeable effect on cognitive function or withdrawal symptoms. Similarly, differential treatment effects due to a 3.3mg caffeine difference between CGA and decaffeinated green blend coffee are even less likely. To the best of our knowledge no previous studies have investigated the effects of caffeine on withdrawal with doses lower than 10mg. For example, in a study by Heatherley et al. [30], caffeine withdrawal effects following 8 hours of abstinence were ameliorated using 1.2 mg/kg caffeine, which in a 70kg person is equivalent to 84mg caffeine. Due to difficulties in removing caffeine entirely from coffee, most decaffeinated coffees will contain at least some proportion of caffeine. For instance, a study by McCusker et al. [31] reported caffeine in the range 0-13.0mg/480g serve amongst 10 different decaffeinated products. However, it is an important consideration that in future studies concerning non-caffeine constituents as little caffeine as possible should be included in the decaffeinated preparations.

### Are the positive effects of decaffeinated green blend coffee attributed to the CGAs?

When compared to decaffeinated green blend coffee, CGA produced an acute slowing of reaction time performance, as measured by the slowing of Decision and Movement times on the Jensen Box tasks. Specifically, in comparison to decaffeinated green blend coffee, CGA was found to be associated with worse performance on both the 2-choice and 4-choice Jensen task with an overall trend towards slower Decision Times with CGA compared to green blend across both time points. Furthermore, in the 4-choice task, there was a trend towards slower Decision Time at 40 minutes after consuming CGA in comparison to decaffeinated green blend coffee. This was corroborated with an overall trend towards slower Movement Time with CGA compared to green blend on the 4-choice Jensen task, irrespective of time and also at 120 minutes specifically. These results suggest that improvements in cognitive performance observed with decaffeinated Green Blend are not likely to be attributed to the CGAs. 

Following cognitive testing, decaffeinated green blend Coffee was found to significantly increase Bond-Lader Alertness ratings in comparison to CGA irrespective of time. Furthermore, when time was factored, decaffeinated green blend coffee increased Alertness at 40 minutes (trend-level) and at 120 minutes (significant) compared to CGA. Corroborating the findings from the Bond-Lader Alert scale, for the Caffeine-VAS Alert scale (post- cognitive testing) subjects were also significantly more alert after receiving green blend compared to CGA across both time points. In addition, subjects receiving CGA were found to be significantly less relaxed (Caffeine-VAS scale) at 120 minutes (post- cognitive testing) in comparison to those receiving decaffeinated green blend. Finally, for the Caffeine-VAS Mental Fatigue scale, at 120 minutes (post- cognitive testing) there was a trend towards higher ratings of mental fatigue following CGA consumption in comparison to Green Blend. 

In summary, compared to placebo, both CGA and decaffeinated green blend coffee tended to improve symptoms of tiredness, headaches and jitteriness. However, no significant differences were found between CGA and decaffeinated green blend suggesting that it could be the CGAs found in the green blend coffee that are attributed to these effects. However, the acute improvement in subjective ratings of Alertness that was observed with decaffeinated green blend was notably absent following CGA consumption. In a number of other mood scales, specifically Mental Fatigue and Relaxation, green blend also outperformed CGA. Taken together these findings suggest the CGAs may not be completely responsible for the proposed anti-fatigue effect found with decaffeinated green blend coffee and that other compounds in decaffeinated green blend coffee are also contributing to the positive mood and cognitive effects. While subtle differences exist in the Chlorogenic acid profile of decaffeinated green blend and the CGA treatment in the current study (see Table 1), it appears unlikely that 1-30 mg differences in specific CGA components as part of a 6 gram mixture would cause noticeable effects on cognition. A more parsimonious explanation is that during the roasting of green coffee beans, unique compounds are produced in addition to CGAs which may also have acute effects on cognition. Quinides are one such component produced during roasting that may potentially modulate cognition actuely via effects on blood glucose levels [32]. However, other changes in both the anti-oxidant and anti-inflammatory profiles of coffee blends due to total phenols and trigonelline content have also been reported during roasting [33,34]. In future studies, investigation into the acute cognitive effects of coffee constituents in addition to chlorogenic acids are also warranted.

## Conclusions

Whilst neither the decaffeinated green blend coffee or CGA were found to have significant effects on the primary outcome measure of RVIP accuracy when compared to placebo, a number of significant treatment effects were found amongst the secondary outcome measures. No evidence was found for beneficial acute cognitive effects associated with CGA in the current study. Rather, CGA was found to only have negative effects on cognitive performance (specifically, attention (RVIP task), speed of processing (IT task) and reaction time performance (Jensons Box task) when compared to both placebo and decaffeinated green blend coffee. In contrast, compared to placebo, there was some evidence to suggest that decaffeinated green blend coffee may bring about some improvements to attention and reaction time performance, as measured by the N-Back and Jensen Box task, respectively. The contrast in findings regarding CGA and decaffeinated green blend coffe suggest that any improvements to cognitive performance that were observed with decaffeinated green blend coffee are most likely not attributable to the CGAs. 

In terms of mood, the results are more complicated to interpret. CGA was found to improve subjective reports of tiredness, headaches and jitteriness, yet reduce feeling of content, when compared to placebo. These improvements were similarly observed with decaffeinated green blend coffee when compared to placebo. However, no significant differences were found when comparing CGA and decaffeinated green blend coffee directly. Thus, suggesting that it could be the CGAs found in the green blend coffee that are attributed to these positive mood effects, which could most parsimoniously be associated with the amelioration of caffeine withdrawal symptoms. In contrast, decaffeinated green blend coffee was found to increase subjective reports of alertness when compared to both placebo and CGA with small to moderate effect size, suggesting that there are compounds in coffee other than CGAs present in decaffeinated green blend coffee which may exert an alerting effect.. This putative green blend anti-fatigue effect is further corroborated with the increased reports of relaxation and a reduction in feelings of mental fatigue when compared to CGA. Thus taken together, the results suggest that any improvements in cognitive function that were found to be associated with decaffeinated green blend coffee are most likely not attributable to the CGAs. Whilst the decaffeinated green blend-related improvements in mood appear to some extent to be attributable to the CGA content, it appears likely that there are also other compounds in green blend coffee that are contributing to the overall positive cognitive and mood effects.

There are several recommendations for future research based on the results and analyses of the present study. In consideration of the large number of trend-level findings after corrections for multiple comparisons were made, it is apparent that the present study was under-powered. For this reason it will be important to replicate certain aspects of the current study using a larger sample – with power calculations based on a considerably smaller effect size. In consideration of the greater number of treatment effects that were found regarding the visual analogue mood scales in relation to the cognitive tests it would also be appropriate to review the selection of cognitive tests in future studies. It is a distinct possibility that with an acute subtle effect on brain function such as that associated with varying coffee constituents, mood effects are easier to capture than cognitive improvements. In the current study it was encouraging to see that two of the most sensitive tests, the Jensen Box apparatus and the N-Back task, provided the most consistent evidence of cognitive changes. The inclusion of further tests that are of a similar nature to the Jensen Box Task and N-Back task would perhaps maximise the probability of capturing subtle cognitive changes in future. Another issue is that individuals with varying Body Mass Indexes (BMI) may have faster or slower rates of metabolism of the active constituents. For this reason it would be worthwhile for future research to consider administering doses that are adjusted according to the subjects’ BMI. In a future study a dose-escalation design using at least two differing levels of CGAs could be beneficial to better understand the dose-response characteristics associated with CGAs in coffee. Finally, potential for acute cognitive effects associated with coffee constituents other than chlorogenic acid, particularly those produced during the roasting process, also warrants further investigation.

### Data availability

The data for the current study is freely available on request by contacting the corresponding author Prof Con Stough: cstough@swin.edu.au.

## Supporting Information

Checklist S1 **CONSORT Checklist.**
(DOC)

Protocol S1 **Trial Protocol.**
(DOC)
