# Supplementary material for: A Randomised Placebo-Controlled Trial to Differentiate the Acute Cognitive and Mood Effects of Chlorogenic Acid from Decaffeinated Coffee
Source: PLoS One. 2013 Dec 9;8(12):e82897. doi: 10.1371/journal.pone.0082897 (PMC3857311; doi:10.1371/journal.pone.0082897)
Supplement: Protocol S1 — Trial Protocol. (DOC) [file pone.0082897.s002.doc]

**Study Protocol**

**Trial No. 10.09.NRC**

***Final version 4, 26^th^ May 2011***

**Integrating amendment 1&2, 26^th^ May 2011**

**The acute effects of chlorogenic acid on cognitive function and mood in healthy older adults**

Coffee and cognition beyond caffeine

RESPONSIBLE PERSONNEL

| Principal Investigator | Professor Con Stough  Brain Sciences Institute  Swinburne University of Technology  400 Burwood Road,  Hawthorn, Victoria 3122  AUSTRALIA  Tel: +61 3 9214 8167  Fax: +61 3 9214 5230 E-mail: [cstough@swin.edu.au](mailto:cstough@swin.edu.au) | ………..........……….…  Date  .......................................  Signature |
| --- | --- | --- |
| Co-Investigator(s) | Professor Andrew Scholey  Brain Sciences Institute  Swinburne University of Technology  400 Burwood Road  Hawthorn, Victoria 3122  AUSTRALIA  Tel: +61 3 9214 8932  Fax: +61 3 9214 5525 E-mail: [ascholey@swin.edu.au](mailto:cstough@swin.edu.au) | …………………………  Date  .....................................  Signature |
| Sponsor | Professor Peter Van Bladeren  Director  Nestlé Research Centre  PO Box 44 Vers-chez-les-Blanc  CH-1000 Lausanne 26  SWITZERLAND  Tel: +41 (21) 785 8801  Fax: +41 (21) 785 8555 E-mail:[peter.van-bladeren@rdls.nestle.com](mailto:peter.van-bladeren@rdls.nestle.com) | ………………………….  Date  .....................................  Signature |
| Project Leader | Beata Silber, Ph.D.  Cognitive Sciences Group  Nestlé Research Centre  PO Box 44 Vers-chez-les-Blanc  CH-1000 Lausanne 26  SWITZERLAND  Tel: +41 21 785 9242  Fax: +41 21 785 8544 E-mail: [beata.silber@rdls.nestle.com](mailto:beata.silber@rdls.nestle.com) | …………………………  Date  .....................................  Signature |
| Statistical Design and Analysis (SUT) | Professor Con Stough  Brain Sciences Institute  Swinburne University of Technology  400 Burwood Road,  Hawthorn, Victoria 3122  AUSTRALIA  Tel: +61 3 9214 8167  Fax: +61 3 9214 5230 E-mail: [cstough@swin.edu.au](mailto:cstough@swin.edu.au) | …………………………  Date  .....................................  Signature |
| Statistical Design and Analysis (NRC) | Dr Rajat Mukherjee  Applied Mathematics Group  Nestlé Research Centre  PO Box 44  1000 Lausanne 26  SWITZERLAND  Phone: + 41 21 785 8227  Fax: + 41 21 785 8556  E-mail: Rajat.Mukherjee@rdls.nestle.com | …………………………  Date  .....................................  Signature |
| Clinical Project Manager (NRC) | Emma Wynn  Nestlé Research Centre  PO Box 44 Vers-chez-les-Blanc  CH-1000 Lausanne 26  SWITZERLAND  Tel: +41 21 785 8389  Fax: +41 21 785 8556  Email: Emma.Wynn@rdls.nestle.com | …………………………  Date  .....................................  Signature |

STUDY PARTICIPANTS

| Sub-Investigator | Marni Kras  Brain Sciences Institute  Swinburne University of Technology  400 Burwood Road,  Hawthorn, Victoria 3122  AUSTRALIA  Tel: +61 3 9214 5094  Fax: +61 3 9214 5525 E-mail: MKRAS@groupwise.swin.edu.au |  |
| --- | --- | --- |
| Sub-Investigator | Vanessa Cropley, Ph.D.  Brain Sciences Institute, Swinburne University of Technology  John Street, Hawthorn,  Victoria 3122  AUSTRALIA  Tel: +61 3 9214 8291  Fax: +61 3 9214 5525 E-mail: [VCropley@swin.edu.au](mailto:rcroft@swin.edu.au) |  |
| Scientific Advisor | Jeroen Schmitt, Ph.D.  Head of Cognitive Sciences  Nestlé Research Centre  PO Box 44  1000 Lausanne 26  SWITZERLAND  Tel: +41 21 785 8167  Fax: +41 21 785 8544  E-mail: jeroen.schmitt@rdls.nestle.com |  |
| Medical Advisor (NRC) | Dr Maurice Beaumont  Clinical Evaluation Group  Nestlé Research Centre  PO Box 44  1000 Lausanne 26  SWITZERLAND  Tel: +41 21 785 8054  Fax: +41 21 785 8556  E-mail: Maurice.Beaumont@rdls.nestle.com |  |
| Clinical Data Manager | Bernard Decarli  Nestlé Research Centre  Vers-chez-les-Blanc, PO Box 44  CH-1000 Lausanne 26  Switzerland  Tel: +41 21 785 8807  Fax: +41 21 785 8925  Bernard.Decarli@rdls.nestle.com |  |

| **TABLE OF CONTENTS** |
| --- |

1 SUMMARY/SYNOPSIS [7](#__RefHeading___Toc504652701)

2 INTRODUCTION [10](#__RefHeading___Toc504652702)

3 OBJECTIVES OF THE TRIAL [10](#__RefHeading___Toc504652703)

3.1 Main objective [10](#__RefHeading___Toc504652704)

3.2 Secondary objectives [11](#__RefHeading___Toc504652705)

4 TRIAL DESIGN [11](#__RefHeading___Toc504652706)

4.1 Type of trial [11](#__RefHeading___Toc504652707)

4.2 Subjects, groups and centers [11](#__RefHeading___Toc504652708)

4.3 Duration of subject participation [11](#__RefHeading___Toc504652709)

5 Study population [11](#__RefHeading___Toc504652710)

5.1 Description [11](#__RefHeading___Toc504652711)

5.2 Subject inclusion criteria [11](#__RefHeading___Toc504652712)

5.3 Subject exclusion criteria [11](#__RefHeading___Toc504652713)2

6 TREATMENT OF SUBJECTS [12](#__RefHeading___Toc504652714)

6.1 Product description [12](#__RefHeading___Toc504652715)

6.1.1 Composition [12](#__RefHeading___Toc504652716)

6.1.2 Form and dosage [12](#__RefHeading___Toc504652717)

6.1.3 Quality control [13](#__RefHeading___Toc504652718)

6.1.4 Packaging and labeling [13](#__RefHeading___Toc504652719)

6.1.5 Blinding technique [13](#__RefHeading___Toc504652720)

6.2 Treatment administration [13](#__RefHeading___Toc504652721)

6.2.1 Amount, dose, concentration, frequency [13](#__RefHeading___Toc504652722)

6.2.2 Route of administration [13](#__RefHeading___Toc504652723)

6.2.3 Subject compliance [13](#__RefHeading___Toc504652724)

6.3 Concomitant diet and treatment [13](#__RefHeading___Toc504652725)

6.3.1 Permitted concomitant diets/treatments/medications [13](#__RefHeading___Toc504652726)

6.3.2 Unauthorized concomitant diets/treatments/medications [14](#__RefHeading___Toc504652727)

6.4 Product handling [14](#__RefHeading___Toc504652728)

6.4.1 Storage and distribution [14](#__RefHeading___Toc504652729)

6.4.2 Product accountability and reconciliation [14](#__RefHeading___Toc504652730)

7 Definition of measures [14](#__RefHeading___Toc504652731)

7.1 Primary outcome measure: Attention [14](#__RefHeading___Toc504652732)

7.2 Secondary outcome measures: other cognitive tasks [14](#__RefHeading___Toc504652733)

7.3 Secondary outcome measures: Mood scales 16

7.4 Other measures [16](#__RefHeading___Toc504652734)

8 Conduct of the trial [16](#__RefHeading___Toc504652735)

8.1 Study plan, flow chart [16](#__RefHeading___Toc504652736)

8.2 Time schedule [18](#__RefHeading___Toc504652737)

8.3 Subject recruitment [18](#__RefHeading___Toc504652738)

8.4 Baseline [18](#__RefHeading___Toc504652739)

8.5 Visits [19](#__RefHeading___Toc504652740)

8.6 Final Visit 20

8.7 Biological samples [20](#__RefHeading___Toc504652742)

8.8 Follow-up [21](#__RefHeading___Toc504652743)

8.9 Breaking of the code [21](#__RefHeading___Toc504652744)

8.10 Data management 21

8.10.1 Data collection 21

8.10.2 Computerized data entry 21

8.10.3 Computerized edit checks 21

8.10.4 Audit trail 21

9 Statistical aspects and considerations [21](#__RefHeading___Toc504652748)

9.1 Background [22](#__RefHeading___Toc504652749)

9.2 Effects to be estimated [22](#__RefHeading___Toc504652750)

9.3 Sample size calculation [23](#__RefHeading___Toc504652751)

9.4 Randomization [23](#__RefHeading___Toc504652752)

9.5 Statistical analysis [23](#__RefHeading___Toc504652753)

9.5.1 Management of subject withdrawal [23](#__RefHeading___Toc504652754)

9.5.2 Intention to treat analysis [23](#__RefHeading___Toc504652755)

9.5.3 Per protocol analysis [23](#__RefHeading___Toc504652756)

9.5.4 Interim analysis [23](#__RefHeading___Toc504652757)

10 Handling of adverse events [24](#__RefHeading___Toc504652758)

10.1 Definition of adverse events [24](#__RefHeading___Toc504652759)

10.2 Reporting and documentation of adverse events [24](#__RefHeading___Toc504652760)

10.3 Follow-up [24](#__RefHeading___Toc504652761)

11 Legal and ethical prerequisites [25](#__RefHeading___Toc504652762)

11.1 Legal requirements [25](#__RefHeading___Toc504652763)

11.2 Ethical aspects [25](#__RefHeading___Toc504652764)

11.2.1 Protection of the subject’s confidentiality [25](#__RefHeading___Toc504652765)

11.2.2 Informed consent [25](#__RefHeading___Toc504652766)

11.2.3 Ethics committee approval [25](#__RefHeading___Toc504652767)

11.2.4 Declaration of Helsinki [25](#__RefHeading___Toc504652768)

12 Quality assurance [25](#__RefHeading___Toc504652769)

12.1 GCP (Good Clinical Practice) [25](#__RefHeading___Toc504652770)

12.2 Internal quality control [25](#__RefHeading___Toc504652771)

13 Agreements [25](#__RefHeading___Toc504652772)

13.1 Monitoring [25](#__RefHeading___Toc504652773)

13.2 Responsibilities of investigator [26](#__RefHeading___Toc504652774)

13.3 Responsibilities of sponsor [26](#__RefHeading___Toc504652775)

13.4 Termination of study [27](#__RefHeading___Toc504652776)

14 References [28](#__RefHeading___Toc504652777)

15 Appendices [30](#__RefHeading___Toc504652778)

| Appendix 1 | Treatment Labels |
| --- | --- |
| Appendix 2 | List of foods to avoid 24 hours prior to experimental sessions |
| Appendix 3 | Mood Visual Analogue Scales |
| Appendix 4 | Flow chart |
| Appendix 5 | Adverse events handling and AE Report Form |
| Appendix 6 | Declaration of Helsinki |

# SUMMARY/SYNOPSIS

| The acute effects of chlorogenic acid on cognitive function  and mood in healthy older adults | | | | | |
| --- | --- | --- | --- | --- | --- |
| *Principal Investigator:* Prof. Con Stough | | | | | |
| *Study center(s):* Swinburne University of Technology, Melbourne, Australia | | | | | |
| *Objectives:* The primary objective is to evaluate whether an acute dose of chlorogenic acid (CGA) improves cognitive function and mood in healthy older adults.  Secondary objectives are to:   1. Evaluate whether it is the CGAs found in decaffeinated coffee that are attributed to the cognitive and mood enhancing effects; 2. Replicate the positive acute effects of 6 grams decaffeinated coffee on attention and mood; 3. Evaluate whether an acute dose of CGA and decaffeinated coffee improves cerebral blood flow, and whether modulations to cerebral blood flow are associated with CGA/decaffeinated coffee-related modulations to cognitive performance and mood | | | | | |
| *Outcome:*  *Primary outcome measure: Attention*  Rapid visual information processing task (accuracy) - measure of sustained attention. This task also loads heavily on working memory.  *Secondary outcome measures:*  *Other cognitive tests*   - Rapid visual information processing task (as above) - reaction time and false alarm rate - Inspection Time task - measure of visual information processing speed - Serial sevens - measure of concentration and working memory - Serial Threes - measure of concentration and working memory - Jenson Box reaction time task – measure of reaction time performance whereby decision time and movement time are separated - N-back task – measure of sustained and selective attention and impulsivity - Emotional face recognition task - measure of emotional processing and attention   *Mood scales*   - Bond & Lader Visual Analogue Scales (Bond & Lader, 1974) – subjective measure of mood and alertness - Caffeine Research Visual Analogue Scales - subjective assessment of feelings of "relaxed", "alert", "jittery", "tired", "tense", "headache", overall mood", and "mental fatigue".   *Other*   - - Brain blood doppler flow   Cognitive performance and mood will be assessed at baseline, 40 minutes and 120 minutes post treatment consumption. Brain blood doppler flow will be measured at baseline and 90 minutes post treatment consumption. Blood samples will be obtained at baseline (training session day), 10 minutes, 90 minutes, and 160 minutes post treatment consumption for bioavailability analyses.  Note: outcome measures that were found to be modulated with the specially processed decaffeinated coffee in the previous acute clinical trial will be administered in the present study. | | | | | |
| *Design:* This is an acute cross-over, placebo-controlled, double blind, randomized, single centre, clinical trial with three treatment conditions: chlorogenic acid, decaffeinated coffee and placebo. | | | | | |
| *Number of patients(to be enrolled / to be analysed):* 66/58 healthy older adults | | | | | |
| *Description of subjects and main criteria for inclusion:* Healthy older adults (male and female) aged 50+. Participants must be light to moderate coffee drinkers (i.e. with caffeine), where subjects drink no more than 8 cups of coffee a week. No existing or pre-existing physical or neurological conditions, no history of psychiatric, cardiac, endocrine, gastrointestinal, or bleeding disorders, not taking any medication that could potentially affect the outcome of the study (i.e. psychoactive medication) including drugs, excessive amounts of alcohol, non-smokers, not on a potassium reduced diet, not under treatment with spironolactone-like anti-diuretics, aldosteron-receptors antagonists or angiotensin II- antagonists and no food allergies. | | | | | |
| *Product(s) to be tested:*  Chlorogenic acid in placebo matrix (maltodextrin and CHE mixed with coffee flavour and colour); decaffeinated coffee; and placebo (maltodextrin and CHE mixed with coffee flavour and colour). | | | | | |
| *Amount, dosage, route of administration, duration of treatment:* - 540 mg chlorogenic acid mixed in a placebo matrix (5460mg maltodextrin and CHE mixed with coffee flavour and colour). The CGA is equivalent to the level found in 3 servings of a specially processed decaffeinated coffee (the chlorogenic acid will contain a similar profile to the specially processed decaffeinated coffee administered in the acute study);   - decaffeinated coffee (6g); - placebo (6 g maltodextrin and CHE based with coffee flavour, colour and aroma).   All treatments will be administered orally as a hot coffee beverage which must be drunk within 15 minutes. Participants will consume each of the coffee beverages on separate testing days. Experimental testing sessions will be separated by at least a one week washout period. | | | | | |
| ***Study plan:*** | **V_0_** | **V_1_** | **V_2_** | **V_3_** |  |
| *Weeks* | 0  Training session | 2 | 3 | 4 |  |
| Informed consent | X |  |  |  |  |
| History / subjects data | X |  |  |  |  |
| Cognitive tests | X | X | X | X |  |
| Subjective ratings | X | x | X | X |  |
| Brain Doppler blood flow |  | x | x | x |  |
| Blood sampling | X | x | x | x |  |
| Treatment |  | x | x | x |  |
| Safety assessment | X | x | x | x |  |

# INTRODUCTION

Coffee is one of the most widely consumed beverages throughout the world. The physiological and behavioural effects of coffee (with caffeine) have been extensively studied over the past four decades. Research has generally shown caffeine to exert positive effects on cognitive performance such as reaction time, alertness, and vigilance (Haskell et al., 2005, 2008; Smit & Rogers, 2000; Frewer & Lader, 1991; Lieberman et al., 1987; Swift & Tiplady, 1988; Lorist et al., 1995; Rees et al., 1999).

Previous research has focused on the effects of caffeine on cognition, and coffee is only a source of caffeine. Coffee is not only caffeine. Coffee contains many other compounds, such as chlorogenic acids, caffeic acid, and ferulic acid, which are known to exert powerful antioxidant properties (Natella et al., 2002; Pellegrini et al., 2003; Olthof et al., 2001; Nardini et al., 2002; Yu et al., 2006). As caffeine exerts significant effects on the brain (Kenemans & Lorist, 1995; Ruijter et al., 2000), the potential beneficial effects of non-caffeine constituents in coffee are poorly understood. Furthermore, as the effects of non-caffeine compounds may modulate or even oppose those of caffeine (de Paulis & Martin, 2004), the acute effects of other compounds found in coffee are difficult to observe, except in decaffeinated coffee.

In a recent trial conducted by Swinburne University and Nestlé, it was found that 3 servings of regular decaffeinated coffee had little or no effect on cognitive function and mood in a healthy older population, thus suggesting that coffee compounds found in regular coffee do not modulate cognitive function or mood. However, the results did indicate that 3 servings of a specially processed decaffeinated coffee exerted positive trend-level acute effects on cognition (specifically attention) and mood (increasing alertness and relaxation, and decreasing reports of mental fatigue and headaches). These findings suggest that there are compounds (other than caffeine) in this specially processed decaffeinated coffee that appear to have positive effects on cognitive function and mood that are not found or that are not at the same level as that found in regular coffee. As chlorogenic acids (CGA) are the most abundant family of compounds found in this specially processed decaffeinated coffee, it is possible that the positive cognitive and mood effects observed are attributed to the chlorogenic acids. To further explore this possibility, the present study aims to assess the effect of pure chlorogenic acid on cognitive function and mood in a healthy older population. The chlorogenic acid will be derived from coffee and the dose to be administered will be the same as is found in 6g of this specially processed decaffeinated coffee. To further understand whether it is the CGAs in the specially processed decaffeinated coffee that are attributed to the positive effects, a second aim is to compare the effects of pure CGA with the decaffeinated coffee. If it is the CGAs contributing to the positive effects, it is expected that there should be no difference in cognitive performance and mood between pure CGA and decaffeinated coffee.

Finally, as the results from the initial acute clinical trial are the first indication that a non-caffeinated pure coffee may improve cognitive performance, the study findings need to be replicated and confirmed. Thus, the third aim is to replicate and confirm the previous trend-level findings and show that 6g decaffeinated coffee improves attention and mood in a healthy older population.

# OBJECTIVES OF THE TRIAL

## 3.1 Main objective

The primary objective is to evaluate whether an acute dose of CGA improves cognitive function and mood in healthy older adults.

Cognitive function will be assessed with a battery of standard validated behavioural cognitive tests. Cognitive functions assessed will be attention, working memory, emotional attention, information processing speed, and reaction time performance.

## 3.2 Secondary objectives

The secondary objectives of the research project are:

- Evaluate whether it is the CGAs found in decaffeinated coffee that are attributed to the cognitive and mood enhancing effects;
- Replicate and extend the positive acute effects of 6 grams decaffeinated coffee on attention and mood
- Evaluate whether an acute dose of CGA and decaffeinated coffee improves cerebral blood flow and whether modulations to cerebral blood flow are associated with CGA/decaffeinated coffee-related modulations to cognitive performance and mood

# TRIAL DESIGN

## 4.1 Type of trial

The proposed study is an acute, cross-over, double blind, counter-balanced, placebo-controlled, randomized, single centre, clinical trial with 3 treatments: CGA, decaffeinated coffee, and placebo.

## 4.2 Subjects, groups and centres

The number of subjects to complete the study protocol will be 58 (see 9.3 statistical section). All subjects will complete the 3 treatment conditions. Subject recruitment will be 66 to provide for a 10% drop-out rate.

The study will be a single-centred study, where all data will be collected at the Brain Sciences Institute, Swinburne University, Melbourne, Australia.

## 4.3 Duration of subject participation

The duration of the subject participation will be 3 weeks. Subjects will be tested on three different test days, separated by at least a 1 week washout period. Each experimental session will take approximately 4 hours to complete. Prior to the experimental testing sessions, subjects will undergo one separate training session (approximately 1 hour).

# Study population

## 5.1 Description

Healthy older male and female adults aged 50+ will be eligible to participate in the study.

## 5.2 Subject inclusion criteria

All subjects must comply with all the following inclusion criteria:

- Age 50 and over
- Healthy (i.e. absence of all exclusion criteria) older male and female adults
- Light to moderate coffee drinkers (i.e. with caffeine)
- Drink no more than 16 cups of coffee per week
- Participants must abstain from caffeine-containing foods and beverages, alcohol, no foods containing chlorogenic acids, and only food with low polyphenol content for 24 hours prior to the training session and each testing session.
- Written informed consent obtained

## 5.3 Subject exclusion criteria

Subjects representing one or more of the following criteria are excluded from participation in the study.

- Existing or pre-existing physical or neurological conditions
- History of psychiatric, cardiac, endocrine, gastrointestinal, or bleeding disorders
- Clinically high blood pressure
- Under treatment with spironolactone-like anti-diuretics, aldosteron-receptors antagonists or angiotensin II- antagonists
- Hearing impairment
- Psychoactive medications
- History of substance abuse
- Smoker
- Food allergies
- Potassium reduced diet
- Score <24 on the Mini-Mental State Exam
- Currently participating or having participated in another clinical trial during the last 2 months prior to the beginning of this study

# TREATMENT OF SUBJECTS

## 6.1 Product description

The CGA will be manufactured at Nestlé Product Technology Centre (PTC) in Orbe, Switzerland. The CGA is derived from coffee beans. The instant decaffeinated coffee is non-commercial and will also be manufactured by PTC Orbe. The placebo (also produced in PTC Orbe) will consist of a small dose of maltodextrin and CHE (soluble powder made from rice cereal) mixed with coffee flavour and colour. The placebo will also be used as the coffee matrix for the CGA condition.

### Composition

The instant decaffeinated coffee is made from a mixture of roasted and green beans. The CGA is derived from green coffee beans.

Products to be tested:

- 540mg Chlorogenic acid mixed with placebo (maltodextrin and CHE (soluble powder made from rice cereal) mixed with coffee flavour and colour)

- 6 g decaffeinated instant coffee derived from green and medium roasted beans

- Placebo (maltodextrin and CHE (soluble powder made from rice cereal) mixed with coffee flavour and colour)

Potassium will be added at very small doses during the extraction process of the CGA. Therefore, subjects on a reduced potassium diet should be excluded.

### Form and dosage

All products will be in powder form.

Instructions for correct preparation will be printed on the label in English.

All coffee treatments will be dissolved and administered as a normal hot coffee drink. Prior to administration, one coffee sachet (6g) will be dissolved in 300 ml boiled water. Milk and sacchran can be added by participants to suit their own taste.

### Quality control

Quality control will be performed by Nestlé at the production stage/on product release, at the end of data collection, and at other times as deemed necessary. The expiry date of the product is marked on the packaging.

### Packaging and labeling

Coffee treatments will be packaged into individual sachets for each separate testing session. Each sachet will be labelled with the study protocol number (10.09.NRC), treatment code (A, B, or C), storage conditions, preparation instructions, and expiry date (See Appendix 1 for treatment label).

### Blinding technique

All products will be blinded by PTC Orbe. The products will be labelled with the trial number (10.09.NRC), and will be distinguishable by the letter printed on the label (i.e. A, B, or C). The identity of the specific product will be blind to subjects, support staff, investigators and the clinical project manager. The code will only be known by the manufacturer. A code break envelope will be supplied to the study site so that the code may be broken in case of emergency.

## Treatment administration

### 6.2.1 Amount, dose, concentration, frequency

Subjects will receive equivalent of 3 servings of coffee (one of the three study treatment conditions) immediately after baseline testing is completed. Subjects will consume the coffee orally within 15 minutes. In total, each subject will complete three treatment conditions: CGA (540mg CGA mixed in 5460mg placebo matrix;) instant decaffeinated coffee (6 grams); and placebo (6 grams maltodextrin and CHE mixed with coffee flavour and colour). The coffee beverage will be prepared by the investigator at the study site. Milk and sacchran can be added by participants to suit their own taste. Note that in a previous pharmacokinetic study conducted at Nestlé, no modulations to the bioavailability of Nescafé PROTECT was found when black coffee was mixed with milk (Renouf et al., 2008).

### Route of administration

Oral administration.

### Subject compliance

The product will be consumed under supervision of the investigator/research assistant. Subjects will receive a comprehensive list of foods that must be avoided 24 hours prior to each training and testing session (Appendix 2). Participants will receive a reminder phone call prior to each training and testing day to increase compliance.

## 6.3 Concomitant diet and treatment

Any medication/treatment initiated during the course of the trial must be recorded in the case report form.

### 6.3.1 Permitted concomitant diets/treatments/medications

Medication for the control of cholesterol and blood pressure is accepted. A standard breakfast/lunch will be provided to all subjects.

### 6.3.2 Unauthorized concomitant diets/treatments/medications

No coffee is permitted for 24 hours prior to each training and testing session. In addition, participants must abstain from caffeine-containing foods and beverages, alcohol, no foods containing chlorogenic acids, and only food with low polyphenol content for 24 hours prior to each training and testing session. A list of foods to be avoided will be provided to all participants (Appendix 2). The use of any psychoactive medications is prohibited throughout the study.

## Product handling

### 6.4.1 Storage and distribution

All study products will be supplied by Nestlé without charge to the investigator. The study products will be supplied to the study site after the investigator has obtained approval from the Ethics Committee and applicable regulatory authorities.

The study product will be stored according to specific instructions accompanying the shipment. Dispensing shall be done on a day-to-day basis by the investigator

### Product accountability and reconciliation

All test articles (investigational product and control) received and dispensed by the investigator will be inventoried and accounted for throughout the trial period by the investigator on the corresponding “product distribution list” (to be delivered during the trial launching visit).

The investigator agrees not to supply the test articles to any person except the subjects participating in this trial. Unused product must not be traded. Unused product remaining at completion of the study will be returned via the Nestlé representative or the sponsor can arrange with the investigator for it to be destroyed onsite with a proper certification of destruction.

# Definition of measures

Cognitive testing will consist of selected tests of attention, working memory, emotional attention, information processing speed, and reaction time performance.

## 7.1 Primary outcome measure

Rapid Visual Information Processing (RVIP) task is a test of sustained attention. This task also loads heavily on working memory. Single digits (1-9) are presented continuously in the middle of a computer screen in a semi-random order. Participants are required to press the response button as soon as they detect three consecutive odd or three even digits in ascending order (i.e. 2,4,6; 3,5,7; 4,6,8; 5,7,9). The digits are presented at a rate of 100 digits per minute. Stimulus duration is 600ms with no inter-stimulus interval. There are eight target sequences (i.e. potential correct hits) presented per minute (96 targets in total). The total duration of the task is 12 minutes. The outcome measure is the number of correct hits (accuracy).

## 7.2 Secondary outcomes measures: other cognitive tasks

- - Rapid Visual Information Processing (RVIP) as above. The outcome measure is the mean reaction time for correct hits and false alarm rate.
  - Inspection Time (IT) is a measure of perceptual speed. This task assesses the presentation time that a subject requires to discriminate between two possible stimuli. The task consists of a stimulus with two vertical parallel lines joined at the top by a horizontal line. There are two versions of the stimulus; either the left line is shorter than the right or the right line is shorter than the left. Stimuli are flashed on a computer screen and the participant is instructed to press a key corresponding to the side of the symbol that is shorter. Each stimulus presentation is followed by the presentation of a backward visual mask. This prevents further processing of the stimulus in iconic memory. The speed of stimulus presentation is varied according to the accuracy of the participants’ responses. The length of presentation of the backward visual mask also varies to determine the optimal visual encoding time. The objective is to respond as accurately, rather than as quickly, as possible. The duration of stimulus presentation is varied until an 80% accuracy level is obtained by the participant. This is taken as the outcome measure for speed of visual information processing speed.
  - Extended Serial Sevens is a computerised task measuring concentration and working memory. A random starting number between 800 and 999 will be presented on the computer screen. Participants are required to count backwards in sevens from the given number, as quickly and as accurately as possible. Responses are made using a numeric keyboard. The outcome measures are the total number of subtractions and number of errors. The duration of the task is 2 minutes.
  - Serial Threes is identical to Serial Sevens (see above), except that it requires serial subtractions of threes. The duration of the task is 2 minutes.
  - Emotional Face Recognition task is a measure of emotional processing and attention. This task requires participants to view pictures of facial affect consisting of three basic emotions (happy, sad and neutral) taken from the Ekman Pictures of Affect Series (Ekman and Friesen, 1976). To increase the difficulty of the task, for each actor, happy and sad faces are morphed with their neutral face (50% linear morph) as described previously. For each trial, a picture of a face depicting an emotional or neutral expression will be presented in the middle of the screen. Three emotion labels (happy, neutral, sad) will be presented simultaneously beneath the picture as a reminder to the participant of which response button corresponded to which emotion. The face stimuli will be 12 x 8 cm, and the emotion labels will be presented in a 3.4 x 2 cm text box. Participants will be seated 60 cm from the screen. The facial stimuli will be made up of six actors (3 males and 3 females), with each emotion presented 108 times (18 times by each actor), giving a total of 324 stimuli presentations. Each trial began with a fixation cross in the centre of the screen for about 300 ms, followed by the face (and the emotion labels) for 1200 ms. Participants are required to identify the faces as happy, sad or neutral by pressing a corresponding button on a button box. Participants are instructed to respond as accurately as possible. Immediately before the actual task, participants will perform a practice task consisting of photographs of the two unused actors from the Ekman series. Behavioural outcome measures are percent correct recognition (accuracy).
  - N-back task is a measure of sustained and selective attention and impulsivity. Single digits are presented on the screen. Participants are required to press ‘YES’ or ‘NO’ using a button box, to indicate whether the digit is the same as the *n* previously (e.g. 1-back the previous digit, 3-back the digit 3 previously). The task is scored for speed and accuracy. The duration of the task is approximately 4 minutes.
  - Reaction Time will be assessed with the Jensen box. This is an apparatus that distinguishes decision time and movement time from total reaction time. This apparatus has eight lights which are arranged in a semi-circular configuration. A response button is located adjacent to each light. A "home" button is situated in the centre of the panel. Subjects are required to press the home button until they see a target light and then to release the home button as quickly as possible and to press the response button adjacent to the stimulus light. Decision time (DT) is defined as the time from stimulus onset to the release of the home button, and movement time (MT) as the time from release of the home button to the depression of the stimulus button. Choice is manipulated by varying the number of stimulus alternatives, from 0 (i.e. one light at one possible location) to multiple (i.e. the stimulus may appear in any one of the eight light positions). Participants will be given several practice trials in the eight stimulus (i.e. eight lights) condition so that they can familiarise themselves with the task. DTs of less than 150 ms are discarded as outliers, as it has been argued that physiological limits prevent shorter DTs (Jensen, 1987). DTs over 999 ms will also be discarded and replaced with an additional trial. In addition, all DTs exceeding three SDs above the subject's mean DT are also discarded (Jensen, 1987). The outcome measures are the median, mean and intra-individual variability (*i - average standard deviation) of both DT and MT for all choice, intercept of the DT function across choice and the slope of this function.

## 7.3 Secondary outcome measures: Mood scales

Bond and Lader Visual Analogue Scales (VAS) (Bond & Lader, 1974) is a frequently used self-evaluation mood rating scale (Appendix 3). In total, 16 dimensions of mood are given. The participant is required to mark, on a 100 mm line to what extent the described state is appropriate to him/her at that moment in time. The Bond and Lader VAS discriminates three affective dimensions: alertness, contentment, and calmness.

Caffeine Research Visual Analogue Scales(Appendix 3) consists of seven visual analogue scales ("relaxed", "alert", "jittery", "tired", "tense", "headache", overall mood") that have previously been used in research into the effects of caffeine (Rogers et al., 2003). In addition, a single "mentally fatigued" visual analogue scale was included, as previous research has shown it to be sensitive to a caffeine-glucose drink (Kennedy & Scholey, 2004).

## 7.4 Other measures

- - Doppler Ultrasound measures blood flow both going towards the brain (in the common carotoid artery and internal carotoid artery) and in the brain (in the middle cerbral artery). The outcome varible is mean velocity.
  - Mini-Mental State Examination (MMSE) (Folstein et al., 1975) is a brief 30-point test that is commonly used to screen for dementia. The MMSE evaluates six areas of cognitive function: orientation, attention, immediate recall, short-term recall, language, and the ability to follow simple verbal and written commands. The MMSE is divided into two sections. The first part requires vocal responses to the examiner's questions. The participant is asked to repeat a short phrase after the examiner; to count backward from 100 by 7s; to name the current season and similar brief items. It tests the participants' orientation, memory, and attention. The maximum score for this section is 21. In the second part of the examination, the participant is asked to follow verbal and written instructions, write a sentence spontaneously, and copy a geometric figure. The test is not timed but usually takes less than 10 minutes to complete. Participants will be excluded if they score less than 24 on the MMSE. In an elderly population, a cut-off of 24 provides high sensitivity and specificity (Spreen & Stauss, 1991).
  - Blood samples will be obtained for internal analyses of the bioavailability of various polyphenols such as caffeic acid, ferulic acid, etc. Blood samples will be taken at baseline (during the training session), 10 minutes, 90 minutes, and 160 min post treatment consumption.

# Conduct of the trial

## 8.1 Study plan, flow chart

A flow chart of the testing schedule is outlined in Appendix 4.

A short telephone interview will be conducted will all potential subjects to ensure study criteria are met (i.e. see Section 5 Study Population). Successful volunteers will then attend one training session. Note that prior to the training session subjects will be a given a list of foods that need to be avoided prior to the training session. Specifically, subjects will be asked to abstain from caffeine-containing foods and beverages, alcohol, no foods containing chlorogenic acids, and only food with low polyphenol content for 24 hours prior to each testing session. During the training session, subjects will complete demographics and medical questionnaire, the MMSE, and informed consent will be obtained (if criteria are met). Subsequently, subjects will complete the battery of cognitive tests to familiarize subjects with study procedures and minimize procedural learning effects. A baseline blood sample will be obtained.

Approximately 24 hours prior to each testing session, subjects will receive a reminder telephone call that they must abstain from caffeine-containing foods and beverages, alcohol, no foods containing chlorogenic acids, and only food with low polyphenol content for 24 hours prior to each testing session. Subjects are instructed to arrive at the laboratory well rested (following a normal night’s sleep). At the beginning of each experimental session, bread with various spreads and orange juice will be provided. After the participant finishes eating, subjects will complete the mood visual analogue scales followed by the cognitive tests. The mood scales will be completed a second time immediately after baseline cognitive testing is finished. The battery of tests should take approximately 40 minutes to complete. Subsequently, baseline brain doppler blood flow will be obtained. Subjects will then be administered the equivalent of 3 servings of coffee, which must be consumed within 15 minutes.

Based on a previous pharmacokinetic study conducted at Nestlé, following oral ingestion of Nescafé PROTECT coffee, several polyphenol metabolites were shown to peak in plasma within 2 hours after coffee ingestion (Renouf et al., 2008). Based on these results and the results from the acute clinical decaffeinated coffee trial (08.17.NRC), cognitive testing will begin 40 minutes after treatment consumption.

Ten minutes following treatment consumption the first blood sample will be obtained. Forty minutes following treatment consumption participants will complete the mood visual analogue scales, followed by the cognitive tests, and again the mood visual analogue scales. A second blood sample will be obtained and brain doppler blood flow will be measured (approximately 90 minutes post treatment consumption). Participants will then rest for approximately 15 minutes before the final battery of cognitive and mood tests are administered 120 minutes post treatment consumption. Specifically, 120 minutes following treatment consumption, participants will complete the mood scales, followed by the cognitive test battery, and again the mood scales. A final blood sample will be obtained 160 minutes post treatment consumption.

Participants will complete the experimental session three times. Each experimental session will take approximately 4 hours to complete. Experimental sessions will be separated by at least a one-week washout period.

The time schedule for each experimental session will be as follows:

NOTE: baseline blood measures will be taken during training session. Subjects must adhere to restricted diet prior to training session also (see above)

- 0 - 15 min Breakfast/Lunch served

- 15 - 17 min Mood visual analogue scales

- 17 - 57 min Cognitive testing

- 57 - 60 min Mood visual analogue scales

- 60 - 70 min Brain blood doppler flow

- 70 - 85 min COFFEE CONSUMPTION

- 95 - 105 min Blood sample

- 125 - 127 min Mood visual analogue scales

- 128 - 173 min Cognitive testing

- 173 - 175 min Mood visual analogue scales

- 175 - 190 min Brain doppler blood flow and blood sample

- 205 - 207 min Mood visual analogue scales

- 207 - 250 min Cognitive testing

- 250 – 252 min Mood visual analogue scales

- 252 - 265 min Blood sample

- 265 min Finish

## 8.2 Time schedule

The duration of the study is expected to be approximately 5 months from the beginning of subject recruitment until the final data collection point. Subjects will be scheduled in such a manner that four subjects are tested per day, with a total of 16 per week. Target starting date is October 1 2010.

Timelines from starting date are:

Week Activity

1 - 2 Recruitment and training session subjects 1 - 16

3 - 5 Testing subjects 1 - 16

6 - 7 Recruitment and training session subjects 16 - 32

8 - 10 Testing subjects 16 - 32

11 - 12 Recruitment and training session subjects 32 - 48

13 - 15 Testing subjects 32 – 48

16 - 17 Recruitment and training session subjects 49 - 65

18 - 20 Testing subjects 49 - 65

21 - 25 Database entry and statistical analyses

26 - 29 Report writing

## 8.3 Subject recruitment

Subjects will be recruited by means of posters and advertisements in local newspapers. After volunteers have satisfied the initial inclusion criteria (completed over the telephone), volunteers will be invited to the study site for a training session. During this session, subjects will complete demographics and medical questionnaire, MMSE, and informed consent will be obtained. Baseline blood measure will be obtained during the training session. Volunteers will be enrolled in the study once they have fulfilled all inclusion criteria, present none of the exclusion criteria, and have signed the consent form.

## 8.4 Baseline

Baseline measures will be obtained after a training session (except for blood which will be obtained during the training session) has been completed (V0). Baseline measures will be obtained at the beginning of each testing session and will include the full battery of cognitive tests, subjective mood ratings, brain doppler blood flow.

## 8.5 Visits

**V0 (Training)**

- The investigator will provide information regarding the study procedures, aims, and requirements.
- Medical history and demographics questionnaire will be completed.
- Mini-Mental State Examination (MMSE)
- If all inclusion criteria have been satisfied, informed consent will be obtained.
- Subjects will complete the full battery of cognitive tests and questionnaires to familiarize them with study procedures and minimize procedural learning effects. Specifically:

- Emotional face recognition task

- Jenson Box reaction time task

- Inspection Time task

- Serial sevens

- Serial threes

- N-back task

- Rapid Visual Information Processing task

- Bond and Lader Visual Analogue Scales

- Caffeine Research Visual Analogue Scales

- Blood sample (baseline)

**V1 (Week 2)**

- Safety assessment recorded in CRF (adverse event record)
- Mood visual analogue scales (Bond-Lader Visual Analogue Scales & Caffeine Research Visual Analogue Scales) will be completed six times (immediately before and after cognitive testing at baseline, 40 minutes and 120 minutes post treatment consumption)
- Cognitive testing will be completed three times (baseline, 40 minutes and 120 minutes post treatment consumption). Specifically:

- Emotional face recognition task

- Jenson Box reaction time task

- Inspection Time task

- Serial sevens

- Serial threes

- N-back task

- Rapid Visual Information Processing task

- Bond and Lader Visual Analogue Scales

- Caffeine Research Visual Analogue Scales

- Blood sample (three times: 10, 90 and 160 minutes post treatment consumption)
- Brain doppler blood flow (baseline and 90 minutes post treatment consumption)

**V2 (Week 3)**

- Safety assessment recorded in CRF (adverse event record)
- Mood visual analogue scales (Bond-Lader Visual Analogue Scales & Caffeine Research Visual Analogue Scales) will be completed six times (immediately before and after cognitive testing at baseline, 40 minutes and 120 minutes post treatment consumption)
- Cognitive testing will be completed three times (baseline, 40 minutes and 120 minutes post treatment consumption). Specifically:

- Emotional face recognition task

- Jenson Box reaction time task

- Inspection Time task

- Serial sevens

- Serial threes

- N-back task

- Rapid Visual Information Processing task

- Bond and Lader Visual Analogue Scales

- Caffeine Research Visual Analogue Scales

- Blood sample (three times: 10, 90 and 160 minutes post treatment consumption)
- Brain doppler blood flow (baseline and 90 minutes post treatment consumption)

**V3 (Week 4)**

- Safety assessment recorded in CRF (adverse event record)
- Mood visual analogue scales (Bond-Lader Visual Analogue Scales & Caffeine Research Visual Analogue Scales) will be completed six times (immediately before and after cognitive testing at baseline, 40 minutes and 120 minutes post treatment consumption)
- Cognitive testing will be completed three times (baseline, 40 minutes and 120 minutes post treatment consumption). Specifically:

- Emotional face recognition task

- Jenson Box reaction time task

- Inspection Time task

- Serial sevens

- Serial threes

- N-back task

- Rapid Visual Information Processing task

- Bond and Lader Visual Analogue Scales

- Caffeine Research Visual Analogue Scales

- Blood sample (three times: 10, 90 and 160 minutes post treatment consumption)
- Brain doppler blood flow (baseline and 90 minutes post treatment consumption)

## 8.6 Final Visit

Final visit is V3

## 8.7 Biological samples

One 7ml blood sample will be obtained during the training session (V0) and three 7ml blood samples will be obtained during each experimental session (V1, V2 and V3). Blood samples will be collected in tube with EDTA as anticoagulant (e.g. Vacutainer BD). Immediately after collection, gently invert the tube eight to ten (8-10) times to evenly mix the blood and anticoagulant. Samples can be kept in fridge or on ice until centrifuging (within 2 hours after collection). Centrifuge the tube at room temperature (at 1600xg for Vacutainer from BD) within 30 minutes of blood collection. Carefully remove the cell-free supernatant plasma without disturbing the cell layer. Transfer the plasma in properly labelled polypropylene test tube by making two aliquots. Store the tubes containing the plasma samples at -20°C or below until shipment on dried ice to NRC. The plasma samples (approx 3ml) will be sent to NRC where analyses will be conducted to assess and compare the bioavailability of various polyphenols found in the pure CGA and the decaffeinated coffee and blood glucose levels.

## 8.8 Follow-up

If any subjects are withdrawn early due to an adverse event, the adverse event protocol will be followed (Appendix 5).

## 8.9 Breaking of the code

The investigator will receive one sealed envelope containing the product identification to be opened only in case of medical emergency.

In circumstances where the investigator identifies an urgent need to break the code, the Clinical Project Manager or the Project Leader should be contacted. All attempts to avoid breaking the code (i.e. withdrawal of treatment) should be made. The code break envelope may be used by the investigator directly in true emergencies. In such cases, the rationale must be documented on the corresponding envelope, with immediate notification of the Clinical Project Manager or the Project Leader.

The code break will also be reported in the subject specific case report form (CRF) and events leading to the emergency breaking will be recorded in the serious adverse event (SAE) report form. All envelopes will be retrieved at the end of the study.

## Data management

### 8.10.1 *Data collection*

Collection mode: paper Case Report Form (CRF)

The CRF will contain all information that is recorded manually, such as the demographic and medical data (medical questionnaires, AEs, SAEs unscheduled visits) which will be collected by the investigator during the training session. The results of the Inspection Time task and the brain doppler blood flow will be recorded directly into the CRF’s.

The complete data set for this study will consist of:

- CRF
- Laboratory data
- Electronic files from cognitive tests. If necessary, the format file, contents and other useful information will be discussed directly with the provider of the data.

The CRF’s are an integral part of this protocol. All data required in the protocol and captured by the investigator will be recorded in the CRFs. The case report forms are made of non-carbon required paper (NCR paper) providing two copies of each page.

All other data should be checked for accuracy versus the subject record.

### *Computerized data entry*

With exception to the Inspection Time task, the Jenson Reaction Time Box task, and the mood visual analogue scales, all cognitive data will be automatically recorded on the computer, and subsequently transferred to a computer database file for statistical analyses. Data for the Inspection Time task and the mood visual analogue scales will also be transferred to the computer database file for statistical analyses.

Data will be entered from the CRFs into a computer database (Clintrial^TM^ 4.6 from Phase Forward – website: [http://www.phaseforward.com](http://www.phaseforward.com/)) at the Nestlé Research Centre (NRC). Upon study completion, all original CRFs will be collected by the CRA/monitor and sent to NRC. Copies of all forms remain at the study site.

### *Computerized edit checks*

The Data Manager will program computerized edit checks at least for data on primary outcome in order to detect discrepancies.

Discrepancies will be treated and if necessary sent to the Investigator on a Data Clarification Form for explanations.

### *Audit trail*

The Clinical Data Management System allows an audit of actions performed by users.

# Statistical aspects and considerations

## 9.1 Background

The primary objective suggested for this trial is to evaluate the effect of acute CGA on attention in terms of performance on the Rapid Visual Information Processing (RVIP) task. In particular, the Total Number of correct Hits, the Reaction Time for the Correct Hits and the False Alarm Rate have been specified. We use the data from a previous trial (08.17.NRC) to estimate the effect sizes and to calculate the sample size needed to have 80% power to detect these effects. In particular, we use the 08.17.NRC data from the Placebo and Decaffeinated Coffee from Green Beans (which is abundant in CGA). The RVIP task consists of identifying targets (sets of 3 consecutive odd/even numbers) out of 96 possible targets. Thus theoretically speaking, this is a binomial trial for each subject with size=96. The success rate is estimated (using data from the placebo group in Trail 08.17.NRC) to be p = 63/96 = .656 which is moderate to large. Thus we expect the data to be fairly Normally distributed. A mixed analysis of covariance (ANCOVA) model was fit to the placebo and decaf coffee from green beans data from the 08.17.NRC trial. The normality and homoscedasticity assumptions were checked. The estimated effect size was an increased score of 2.45 points on the RVIP as compared to placebo and the with-in standard deviation was estimated to be 6.52.

## Effects to be estimated

### 9.2.1 Primary Endpoint:

RVIP accuracy score

### 9.2.2 Secondary Endpoint:

The secondary endpoints concerned are:

- Inspection Time – time that target is correctly identified in 80% of trials
- Serial Sevens - total number of subtractions and number of errors
- Serial Threes - total number of subtractions and number of errors
- Emotional face recognition task – percent of correctly recognised emotions
- N-back task - speed and accuracy
- Reaction Time – mean reaction time (ms)
- Bond and Lader Visual Analogue Scales
- Caffeine Research Visual Analogue Scales
- Brain doppler blood flow - mean velocity

Same comparison as primary objective on secondary outcomes:

Assess the effect of CGA vs. placebo on secondary outcomes.

Other comparisons of interest:

Assess the effect of CGA vs. decaffeinated coffee on primary and secondary outcomes.

Assess the effect of decaffeinated coffee vs. placebo on primary and secondary outcomes.

## 9.3 Sample size calculation

With the above estimated effect size and with-in standard deviation, 58 subjects would be needed in the crossover study. With a dropout rate of 10% (as also suggested in 08.17.NRC, 4/43), the total number of subject to be enrolled is 66.

## 9.4 Randomization

The products used in this study will be coded (A, B and C). A randomization schedule will be generated using Williams Design in R 2.6.1 using these codes. The randomization schedule will be made available to the investigator by hard copy.

## 9.5 Statistical analysis

9.5.1 Primary Outcome

As mentioned in section 9.1, we expect the accuracy score on the RVIP to be fairly normally distributed, since the Central Limit Theorem applies here. The proposed analysis is a mixed ANCOVA model that adjusts for the baseline scores. The mixed model here will incorporate a subject specific random intercept term which will accommodate for correlated repeated measurements with-in each subject. The main independent variables will be time, treatment and if necessary their interaction.

During the blind review and prior to data freezing and code breaking, the feasibility of the above proposed analysis plan will be verified using the blinded data. In case the above proposal is deemed not feasible, alternative methods like using normalizing transforms or non-parametric methods will be proposed in the updated final statistical analysis plan.

9.5.2 Secondary Outcome

For the secondary outcomes, similar mixed models will be used. Again the feasibility of such analysis for each outcome will be checked during the blind review and a detailed analysis plan for the secondary outcome will be proposed in the final statistical analysis plan.

### 9.5.1 Management of subject withdrawal

Sample size is adjusted to account for a possible 10% drop out rate. Thus, 66 subjects need to be recruited.

### 9.5.2 Intention to treat analysis

Data from all randomized subjects will be considered in the intention to treat model (for the primary outcome).

### 9.5.3 Per protocol analysis

Per protocol, evaluation will exclude data from subjects in the following conditions:

- Prohibited concomitant treatment
- Subjects without the primary outcome available for Decaffeinated coffee and placebo
- Major violation of inclusion / exclusion criteria

### 9.5.4 Interim analysis

None needed.

# Handling of adverse events

## 10.1 Definition of adverse events

The adverse event is defined as any untoward occurrence in a patient or clinical investigation subject administered an investigational product and which does not necessarily have to have a causal relationship with this treatment.

Adverse events are illnesses, signs or symptoms occurring or worsening, and/or abnormal laboratory findings during the course of the study. Adverse events include occasions when the subjects contact the investigator or their private physician and are examined or given medical direction. They may or may not lead to the withdrawal of the subject from the study.

Adverse events are generally classified as serious or non-serious. See Appendix 5 for classification system.

## 10.2 Reporting and documentation of adverse events

All adverse events occurring during the study will be reported and recorded whether or not they are considered to be non-serious, serious and/or related to the treatment. The following information will be required in each case:

- Subject and date
- Description of event
- Duration
- Frequency
- Intensity
- Seriousness
- Action taken
- Outcome and sequelae
- Relationship to test product

Definitions are included in Appendix 5.

Documentation of all adverse events includes completion of the appropriate section of the case report form (AE).

Documentation of a serious adverse event requires that a separate form (SAE) be completed by the investigators in each case (see Appendix 5).

In addition, the sponsor (via the Project Leader) will be notified by the investigator of serious adverse events. Such events should be reported immediately by phone and followed by a faxed SAE form within 24 hours.

## 10.3 Follow-up

If further information examinations are required to assess the relationship between an adverse event and treatment following the occurrence of the adverse event, all examinations or laboratory findings must be noted with their results in the CRF (section UV) or attached to a follow up file.

# Legal and ethical prerequisites

## 11.1 Legal requirements

Trial will be conducted according to the ICH-guidelines of Good Clinical Practice (GCP) set out by the Swinburne University Research Ethics Committee and the Therapeutics Goods Administration (TGA).

## Ethical aspects

### 11.2.1 Protection of the subject’s confidentiality

Confidentiality of all study participants will be maintained; codes for subject identification will be utilized.

### Informed consent

When the investigator has determined that the subject is an appropriate candidate for the study, the study will be described and explained orally to the subject. The investigator will answer fully all questions. A copy of the information sheet will be given to the subject.

Written, informed consent will be obtained from each subject by the investigator prior to enrolment in the study. The consent form will be signed and dated by the subject and the investigator. The consent form will be completed in three copies: the first copy is kept in the investigator’s file, the second is kept in the subject’s notes, and the third is given to the participant.

No subject will receive treatment before completion of the written informed consent.

### Ethics committee approval

The study protocol will be submitted by the investigator for examination to the Institutional Ethics Committee (IEC). Commencement of the clinical trial is not permitted without written approval of the ethics committee.

The IEC must be notified of all subsequent additions or changes in the study protocol. Notification of the IEC is also required in the event of a SAE during the clinical trial.

### Declaration of Helsinki

This trial will be conducted according to the principles and rules laid down in the Declaration of Helsinki (Appendix 6) and its subsequent amendments.

# Quality assurance

## 12.1 GCP (Good Clinical Practice)

This clinical trial will be conducted following the principles of ICH (International Conference on Harmonization) guideline for Good Clinical Practice and adherence with the applicable regulatory or legal requirements.

## 12.2 Internal quality control

The clinical trial will be conducted according to GCP and Standard Operating Procedures (SOPs) of the Brain Sciences Institute, Swinburne University of Technology, as well as the SOPs of the Nestlé Research Center, Lausanne, Switzerland.

# Agreements

## 13.1 Monitoring

Regular monitoring visits by representatives of the sponsor will be made during the study.

Monitoring will begin with an initial visit prior to study commencement, or shortly after commencement, to clarify all aspects of the protocol and documentation. The purpose of later visits during the implementation period will be to evaluate study progress and adherence to protocol. The CRA/monitor will check CRFs for completeness, clarity and consistency with the information in patients files (source data checking). At the end of the trial the CRA/monitor will make a study closing visit to all sites to ensure that all documentation is complete. In all cases, it is the responsibility of the CRA/monitor to maintain patient confidentiality.

## 13.2 Responsibilities of investigator

The investigators are responsible for the following:

- Obtaining the written and dated approval of the local ethics committee (and other local regulatory agency, if any) prior to the beginning the study
- Selection of participants in accordance with the inclusion and exclusion criteria; obtaining the informed consent of the subject or legal guardian
- Maintain confidentiality of subjects and potential subjects in accordance with the Declaration of Helsinki
- Adherence to the study protocol and the spirit of Good Clinical Practice. If modification becomes necessary, the rationale will be provided in a protocol amendment signed by the investigator and sponsor for submission to the ethics committee.
- Accurate and complete data collection
- During the course of the trial, provide subjects with any newly available information which may be relevant to them
- Identification of adverse events with notification to sponsor, ethics committee and health authorities, as applicable
- Co-operation with monitoring visits, audits and regulatory inspections. Providing direct access to source data and documents
- Investigator may select a second contact at their study center to assist in implementation of the study. However, in all cases the main responsibility with respect to all aspects of this implementation rest with the principal and co-investigators.
- Archiving of the Investigator’s file (including the original signed informed consent forms of all patients) for at least 10 years after the end or the termination of the trial.

## 13.3 Responsibilities of sponsor

The sponsor is responsible for the following:

- Preparation of study products under Good Manufacturing Practice (GMP)
- Product labelling to maintain blinding/masking per protocol
- Delivery of study products to study site with maintenance of records of product shipments
- Distribution of study protocol, case report forms, dietary records and sample collection supplies *(as appropriate)* to clinical sites
- Adherence to study protocol. If modification becomes necessary, the rationale will be provided in a protocol amendment which will be signed by both investigator and sponsor.
- Regular study monitoring
- Obtaining insurance protection
- Archiving of the Study Master File (including the original case report forms) for at least 5 years beyond the expiration date of the last batch.

## 13.4 Termination of study

The study, once commenced, can only be terminated in accordance with Article 15 of the Clinical Trial Agreement between Swinburne and Nestec. Should it prove necessary to discontinue the study permanently prior to completion the sponsors will notify the investigators and additional contacts, including the IRB/IEC, of the rationale. All study relevant documents will then be returned to the sponsor, and the study product will be destroyed or returned.

# References

Bruce, M., Scott, N., Lader, M., Marks, V. (1986). The psychopharmacological and electrophysiological effects of single doses of caffeine in healthy human subjects. British *Journal of Clinical Pharmacology, 22*, 81-7.

de Paulis, T. and Martin, P. R. (2004). Cerebral effects of non-caffeine constituents in roasted coffee. In A. Nehlig (Eds). *Coffee, Tea, Chocolate and the Brain*. Boca Raton, Florida: CRC Press LLC: pp.187-195.

Ekman, P. and Friesen, W. (1976). *Pictures Of Facial Affect*. Palo Alto, CA: Consulting Psychologists Press.

Folstein, M. F., Folstein, S., E., McHugh, P. R. (1975). "Mini-mental state". A practical method for grading the cognitive state of patients for the clinician. *Journal of Psychiatric Research, 12*, 189-98.

Frewer, l. J. and Lader, M. (1991). The effects of caffeine on two computerised tests of attention and vigilance. *Human Psychopharmacology, 6*, 119-28.

Haskell, C. F., Kennedy, D. O., Wesnes, K. A., Scholey, A. B. (2005). Cognitive and mood improvements of caffeine in habitual consumers and habitual non-consumers of caffeine. *Psychopharmacology, 179*, 813-25.

Haskell, C. F., Kennedy, D. O., Milne, A. L., Wesnes, K. A., Scholey, A. B. (2008). The effects of L-theanine, caffeine and their combination on cognition and mood. *Biological Psychology, 77*, 113-22.

Kenemans, J. L. and Lorist, M. M. (1995). Caffeine and selective visual processing. *Pharmacology, Biochemistry, and Behaviour, 52*, 461-71.

Kennedy, D. O. and Scholey, A. B. (2004). A glucose-caffeine 'energy drink' ameliorates subjective and performance deficits during prolonged cognitive demand. *Appetite, 42*, 331-33.

Lieberman, H. R., Wurtman, R. J., Emde, G. G. Roberts, C., Coviella, I. L. G., (1987). The effects of low doses of caffeine on human performance and mood. *Psychopharmacology, 92*, 308-12.

Lorist, M. M., Snel, J., Mulder, G., Kok, A. (1995). Aging, caffeine, and information processing: an event-related potential analysis. *Electroencephalography and Clinical Neurophysiology, 96*, 453-67.

Pellegrini, N., Serafini, M., Colombi, B., Del Rio, D., Salvatore, S., Bianchi, M., Brighenti, F. (2003). Total antioxidant capacity of plant foods, beverages and oils consumed in Italy assessed by three different in vitro assays. *Journal of Nutrition, 133*, 2812-9.

Olthof, M., R., Hollman, P. C. H., Katan, M. B. (2001). Chlorogenic acid and caffeic acid are absorbed in humans. *Journal of Nutrition*, 66-71.

Nardini, M., Cirillo, E., Natella, F., Scaccini, C. (2002). Absorption of phenolic acids in humans after coffee consumption. *Journal of Agricultural and Food Chemistry, 50*, 5735-41.

Natella, F., Nardini, M., Giannetti, I., Dattilo, C., Scaccini, C. (2002). Coffee drinking influences plasma antioxidant capacity in humans. *Journal of Agricultural and Food Chemistry, 50*, 6211-16.

Nawrot, P. Jordan, S. Eastwood, J., Rotstein, J., Hugenholtz, A., Feeley, M. (2003). Effects of caffeine on human health. *Food Additives and Contaminants, 20*, 1-30.

Rees, K., Allen, D., Lader, M. (1999). The influences of age and caffeine on psychomotor and cognitive function. *Psychopharmacology, 145*, 181-88.

Renouf, M. et al. Project Number RDLS-RD080063. Plasma pharmacokinetics of coffee and green tea polyphenols in healthy humans (2008).

Rogers, P. J., Martin, J., Smith, C., Heatherley, S. V., Smit, H. J. (2003). Absence of reinforcing, mood and psychomotor performance effects of caffeine in habitual non-consumers of caffeine. *Psychopharmacology, 167*, 54-62.

Ruijter, J., de Ruiter, M. B., Snel, J. (2000). The effects of caffeine on visual selective attention to colour: An ERP study. *Psychophysiology, 37*, 427-39.

Smit, H. J. and Rogers, P. J. (2000). Effects of low doses of caffeine on cognitive performance, mood and thirst in low and higher caffeine consumers. *Psychopharmacology, 152*, 167-73.

Spreen, O. and Stauss, E. (1991). *A Compendium of Neuropsychological Tests*. New York:

Oxford University Press.

Swift, C. G. and Tiplady, B. (1988). The effects of age on the response to caffeine. *Psychopharmacology, 94*, 29-31.

Yu, G., Maskray, V. Jackson, S. H. D., Swift, C. G., Tiplady, B. (1991).

# Appendices

| Appendix 1 | Treatment Labels |
| --- | --- |
| Appendix 2 | List of foods to avoid 24 hours prior to experimental sessions |
| Appendix 3 | Mood Visual Analogue Scales |
| Appendix 4 | Flow chart |
| Appendix 5 | Adverse events handling and AE Report Form |
| Appendix 6 | Declaration of Helsinki |

Appendix N^o^ 1

tREATMENT LABELS

| **10.09.NRC**  **Instant powder for oral administration**  **Code 1009-A**  **Expire date: ??.2011  Storage: room temperature**  **Quantity: 6g in 300ml water**  *For clinical trial use only, not for sale*  *Produced by PTC Orbe,Switzerland*  *for Nestec Ltd* | **10.09.NRC**  **Instant powder for oral administration**  **Code 1009-B**  **Expire date: ??.2011  Storage: room temperature**  **Quantity: 6g in 300ml water**  *For clinical trial use only, not for sale*  *Produced by PTC Orbe, Switzerland*  *for Nestec Ltd* |
| --- | --- |
| **10.09.NRC**  **Instant powder for oral administration**  **Code 1009-C**  **Expire date: ??.2011  Storage: room temperature**  **Quantity: 6g in 300ml water**  *For clinical trial use only, not for sale*  *Produced by PTC Orbe, Switzerland*  *for Nestec Ltd* |  |

Note: the expiry date will not be known until the CGA is produced.

Appendix N^o^ 2

LIST OF FOODS TO AVOID 24 HOURS PRIOR TO EXPERIMENTAL SESSIONS

**Food and drink to be avoided during 24 hours**

**prior to every testing session.**

| **Drinks**  **AVOID:** | **Drinks that are OK** |
| --- | --- |
| **All** types of tea (e.g. earl grey, green, black, Oolong)  Drinks containing tea (ready to be consumed, instant)  Herb teas  Coffee  Hot chocolate/Cacao/Nesquik/Milo | Hot milk |
| Fruit juice, in particular:  Orange juice  Grape juice  Apple juice | Cordial, lemonade, lemon drink, water |
| All alcohol |  |
| **Fruits, vegetables, nuts**  **AVOID:** | **Foods that are OK** |
| Blackberries | Banana |
| Green and red grapes | Melon |
| Raspberries | Pineapple |
| Strawberries | Pear |
| Blueberries | Carrot |
| Cherries | Pasta |
| **All** citrus fruit such as oranges, grapefruit, lemon, lime, tangerines | Rice |
| Plums |  |
| Apples |  |
| Raisons, Currants |  |
| Olives |  |
| Spinach |  |
| Asparagus |  |
| **All** Nuts, e.g. hazelnuts, walnuts, almonds, peanuts, etc |  |
| **Cereals and Breads**  **AVOID:** | **Foods that are OK** |
| Buckwheat | Cereals containing rice |
| Oats | White bread |
| Barley |
| Corn/Wheat |
| Broad beans |
| Lentils |  |
| **Desserts, sweets, biscuits**  **AVOID:** | **Foods that are OK** |
| Anything containing chocolate e.g. chocolate bars, chocolate, cakes, chocolate biscuits, chocolate cream, chocolate mousse, chocolate ice-cream | Plain yogurt |
| Cereal bars | Honey |
| Jams and frozen fruits |  |
| Nutella |  |
| **Other**  **AVOID:** | **Foods that are OK** |
| Olive oil | Vegetable oil |
| Grape oil |  |

Appendix N^o^ 3

MOOD VISUAL ANALOGUE SCALES

Appendix N^o^ 4

FLOW CHART

**V0**

**V1**

**V2**

1 week

1 week

1 week

**V3**

Wash-out

Wash-out

Wash-out

**Training session**

**Blood sample**

**Mood scales**

**Cognitive test**

**Doppler blood flow**

**Blood sample**

**Mood scales**

**Cognitive test**

**Doppler blood flow**

**Blood sample**

**Mood scales**

**Cognitive test**

**Blood sample**

Experimental session for V 1, 2, and 3

Breakfast

Mood

Cognition

Tx

Mood

Mood

Cognition

Blood Doppler

Mood

Cognition

Blood

Finish

Mood

Blood

Mood

0

15

17

57

70

95

125

128

173

205

207

250

252

265

175

Doppler

60

Appendix N^o^ 5

ADVERSE EVENTS (AE) HANDLING & AE REPORT FORM

## ADVERSE EVENTS HANDLING AND REPORT FORM

**Instructions for “Adverse Events”**

**1- Definition: Adverse event**

**1-1 Adverse event**

An adverse event is defined as any untoward occurrence in a patient or clinical investigation subject administered an investigational product and which does not necessarily have to have a causal relationship with this treatment or usage.

Adverse events are illnesses, signs or symptoms (including an abnormal laboratory finding) occurring or worsening in the course of the study.

Adverse events can be serious or non-serious.

They may or may not lead to the withdrawal of the subject from the study.

All adverse events must be documented and assessed for relationship to the study product.

Investigators must know and record the following information about adverse events:

- Description of AE

- Occurrence of AE prior to study entry

- Date

- Duration

- Intensity

- Frequency

- Relationship to study product

- Seriousness

- Measures taken with regards to study product and medication

- Outcome and sequalae (if applicable).

**1-2 Adverse product reaction.**

A response to a product which is noxious and unintended and which occurs at doses normally used in human for prophylaxis, diagnosis or therapy of disease or for modification of physiological function.

**1-3 Unexpected adverse product reaction**

An adverse reaction, the nature of severity of which is not consistent with the applicable product knowledge’s (e.g., Investigator’s brochure or product description): the product adverse reaction will be classify as unexpected after medical review and medical advisor decision.

**2- Definitions of Seriousness**

**2-1 Serious Adverse Event / serious adverse product reaction**

A serious adverse event or serious adverse product reaction is any untoward medical occurrence at any dose that :

● Results in death.

● Is life-threatening (immediate risk of death).

● Require subject hospitalization or prolongation of existing hospitalization

● Results in persistent or significant disability/incapacity.

● Results in congenital anomaly/birth defect.

● is otherwise judged medically very serious by the Investigator responsible of the study.

Medical judgment should be exercised in determining whether an event is an important medical event. An important medical event may not be immediately life-threatening and/or result in death or hospitalization. However, if it is determined that the event may jeopardize the subject and may require intervention to prevent one of the other outcomes listed in the definition above, the important medical event should be reported as serious.

Examples of such events are intensive treatment in an emergency room or at home for allergic bronchospam, blood dyscrasias or convulsions that do not result in hospitalization or development of product dependency or product abuse.

Laboratory abnormalities identified as critical to safety evaluations should also be reported to the Sponsor.

The investigator will follow up all serious adverse events, regardless of severity, until satisfactory resolution.

**2-2 Non Serious Adverse Event**

All other adverse events not corresponding to the definition of a serious adverse event.

**3- Definitions of assessments**

**3-1 Intensity assessment (only non serious AEs)**

**Mild**: Symptoms hardly perceived, only slight impairment of general well-being.

**Moderate**: Clearly noticeable symptom, but tolerable without immediate relief.

**Severe**: Overwhelming discomfort.

***3-2* Relation with test product / causal relationship**

For all adverse events, sufficient information should be obtained by the investigator to determine causality of the adverse event. The investigator is required to assess causality on the basis of the following criteria:

**Unrelated**: There is an **evident** other explanation for the AE, e.g.,

- The AE is obviously explained by the patient’s disease
- The AE is in accordance with the effect or adverse effect of the concomitant medication
- The AE has occurred already prior to the administration of the study product

**Unlikely relation**: Reasonable temporal relationship with the intake of the study product, **but** there is another plausible explanation for the occurrence of the AE.

**Probable relation**: Reasonable temporal relationship with the intake of the study product **and** plausible reasons point to a causal relationship with the study product

**Definite relation**: Reasonable temporal relationship with the intake of the study product **and**

- There is no other explanation for the AE **and**
- Subsidence or disappearance of the AE on withdrawal of the study product (dechallenge) **and**

- Recurrence of the symptoms on rechallenge

For adverse events with a causal relationship to the investigational product, follow-up by the investigator is required until the event or its sequelae resolve or stabilize at a level acceptable to the investigator, and Nestlé concurs with that assessment.

***3-3* Measures Taken**

**Study product:** This should be recorded as Yes, No or NA. Whether or not the study product was discontinued should be documented. If it had been discontinued, whether or not the product was reintroduced should then be documented.

**Medications:** Any medications given in relation to an adverse event should be recorded on the Concomitant Medication (CM) page of the CRF. In addition, the corresponding number from the CM page of the CRF should be recorded on the Adverse Event form.

**3-4 Outcome**

For all adverse events (serious / non serious), the investigator must pursue and obtain information adequate both to determine the outcome of the adverse event.

**4- Reporting and Documentation**

All adverse events occurring during a study will be reported and recorded whether considered or not as non-serious, serious and/or related or not to the product/ treatment as described in the following sections.

The investigator has to assess whether it meets the criteria for classification as a serious adverse event (see section 2.1 for definition) which requires immediate notification to Nestlé or its designated representative.

**4-1 Reporting period**

Serious adverse events require immediate notification to Nestlé or its designated representative beginning from the time that the subject or legal representative provides informed consent which is obtained prior to the subject’s participation in the study, i.e, prior any study related procedure and/or receiving investigational product.

Adverse events should be recorded on the CRF from the time the subject has taken at least one dose of product.

Any serious adverse event occurring any time after the reporting period must be promptly reported if a causal relationship to investigational product is suspected.

Nestlé will be responsible for the reporting of Serious Adverse Events to Regulatory Authorities and to Ethics Committee(s) for clinical trials conducted in the European Community.

However, if required by applicable local regulations, the Investigator, instead of Nestlé, shall notify the Ethics Committee(s) that a serious adverse event has taken place.

Any SAE occurring within 30 days of the last study product administration will be similarly reported by phone or fax within 24 hours and in writing within 3 days of being informed of the event.

In addition, if required by applicable local regulations, the investigator shall promptly notify the Ethics Committee of any SAE brought to his/her attention by the Sponsor and verify the Ethics Committee acknowledges receipt of the information.

**4-2 Non serious adverse event**

All adverse events must be documented on the appropriate pages of the case report form.

**4-3 Serious adverse event**

The Medical Advisor must be notified of all serious or unexpected adverse events within 48 hours of being informed of its occurrence, per fax or e-mail, using the SAE form (2 pages). Notification does not depend on whether there is a connection to the study product or not.

If the investigator does not become aware of the occurrence of a serious adverse event immediately (e.g subject hospitalized elsewhere), the investigator has to report the event on a SAE form within 48 hours after learning of it and document the time of his/her awareness of the adverse event.

**5- Expedited reporting / Time frames**

**5-1 Serious, Unexpected adverse product reactions**

All adverse prodcut reaction that are both serious and unexpected are subject to expedited reporting. This applies to reports from spontaneous sources and from any type of clinical or epidemiological investigation, independent of design or purpose.

**5-1-1 Fatal or life threatening Unexpected adverse product reaction**

Fatal or life threatening unexpected adverse product reactions occurring in clinical investigations qualify for very rapid reporting, regulatory agencies should be notified as soon as possible but not later than 7 calendars days after first knowledge by the sponsor that a case qualifies, followed by as complete a report as possible within 8 additional calendar days.

**5-1-2 All other Serious, Unexpected adverse product reactions**

Serious, unexpected reactions (ADRs) that are not fatal or life-threatening must be filed as soon as possible but not later than 15 calendar days after first knowledge by the sponsor that the case meets the minimum criteria for expedited reporting.

**5-2 Serious expected adverse product reaction**

Expedited reporting of reactions that are serious but expected will ordinarily be inappropriate. Expedited reporting is also inappropriate for serious events from clinical investigations that are considered not related to study product, whether the event is expected or not.

**SERIOUS ADVERSE EVENTS**

**Please fax to:**

**Dr Maurice Beaumont, Medical Advisor**

**And Emma Wynn, Clinical Project Manager**

**Clinical Evaluation Group**

**Nestlé Research Centre, Switzerland**

**Fax: +41 21 785 85 56**

All serious adverse events must also be documented on the appropriate pages of the case report form.

**5 Follow up**

In the case of a serious adverse events persisting beyond trial termination, a follow up visit may be required.

Further, in the event that additional analyses are required for the evaluation of a potential cause-effect relationship between the study product and the adverse event,

Follow-up by the investigator is required until the event or its sequelae resolve or stabilize at a level acceptable to the investigator and nestlé concurs with that assessment.

All corresponding examinations and laboratory analyses and their results will be documented in the case report forms (section UV) or in an attached file.

Appendix N^o^ 6

DECLARATION OF HELSINKI

| WORLD MEDICAL ASSOCIATION DECLARATION OF HELSINKI  Ethical Principles for Medical Research Involving Human Subjects   |  |  | | --- | --- | |
| --- | --- | --- |
| Adopted by the 18th WMA General Assembly, Helsinki, Finland, June 1964, and amended by the: 29th WMA General Assembly, Tokyo, Japan, October 1975 35th WMA General Assembly, Venice, Italy, October 1983 41st WMA General Assembly, Hong Kong, September 1989 48th WMA General Assembly, Somerset West, Republic of South Africa, October 1996 52nd WMA General Assembly, Edinburgh, Scotland, October 2000  53th WMA General Assembly, Washington 2002 (Note of Clarification on paragraph 29 added) 55th WMA General Assembly, Tokyo 2004 (Note of Clarification on Paragraph 30 added) 59th WMA General Assembly, Seoul, October 2008   1. INTRODUCTION    1. The World Medical Association (WMA) has developed the Declaration of Helsinki as a statement of ethical principles for medical research involving human subjects, including research on identifiable human material and data.   The Declaration is intended to be read as a whole and each of its constituent paragraphs should not be applied without consideration of all other relevant paragraphs.   - 1. Although the Declaration is addressed primarily to physicians, the WMA encourages other participants in medical research involving human subjects to adopt these principles.   2. It is the duty of the physician to promote and safeguard the health of patients, including those who are involved in medical research. The physician's knowledge and conscience are dedicated to the fulfilment of this duty.   3. The Declaration of Geneva of the WMA binds the physician with the words, "The health of my patient will be my first consideration," and the International Code of Medical Ethics declares that, "A physician shall act in the patient's best interest when providing medical care."   4. Medical progress is based on research that ultimately must include studies involving human subjects. Populations that are underrepresented in medical research should be provided appropriate access to participation in research.   5. In medical research involving human subjects, the well-being of the individual research subject must take precedence over all other interests.   6. The primary purpose of medical research involving human subjects is to understand the causes, development and effects of diseases and improve preventive, diagnostic and therapeutic interventions (methods, procedures and treatments). Even the best current interventions must be evaluated continually through research for their safety, effectiveness, efficiency, accessibility and quality.   7. In medical practice and in medical research, most interventions involve risks and burdens.   8. Medical research is subject to ethical standards that promote respect for all human subjects and protect their health and rights. Some research populations are particularly vulnerable and need special protection. These include those who cannot give or refuse consent for themselves and those who may be vulnerable to coercion or undue influence.   9. Physicians should consider the ethical, legal and regulatory norms and standards for research involving human subjects in their own countries as well as applicable international norms and standards. No national or international ethical, legal or regulatory requirement should reduce or eliminate any of the protections for research subjects set forth in this Declaration.  1. BASIC PRINCIPLES FOR ALL MEDICAL RESEARCH    1. It is the duty of physicians who participate in medical research to protect the life, health, dignity, integrity, right to self-determination, privacy, and confidentiality of personal information of research subjects.    2. Medical research involving human subjects must conform to generally accepted scientific principles, be based on a thorough knowledge of the scientific literature, other relevant sources of information, and adequate laboratory and, as appropriate, animal experimentation. The welfare of animals used for research must be respected.    3. Appropriate caution must be exercised in the conduct of medical research that may harm the environment.    4. The design and performance of each research study involving human subjects must be clearly described in a research protocol. The protocol should contain a statement of the ethical considerations involved and should indicate how the principles in this Declaration have been addressed. The protocol should include information regarding funding, sponsors, institutional affiliations, other potential conflicts of interest, incentives for subjects and provisions for treating and/or compensating subjects who are harmed as a consequence of participation in the research study. The protocol should describe arrangements for post-study access by study subjects to interventions identified as beneficial in the study or access to other appropriate care or benefits.    5. The research protocol must be submitted for consideration, comment, guidance and approval to a research ethics committee before the study begins. This committee must be independent of the researcher, the sponsor and any other undue influence. It must take into consideration the laws and regulations of the country or countries in which the research is to be performed as well as applicable international norms and standards but these must not be allowed to reduce or eliminate any of the protections for research subjects set forth in this Declaration. The committee must have the right to monitor ongoing studies. The researcher must provide monitoring information to the committee, especially information about any serious adverse events. No change to the protocol may be made without consideration and approval by the committee.    6. Medical research involving human subjects must be conducted only by individuals with the appropriate scientific training and qualifications. Research on patients or healthy volunteers requires the supervision of a competent and appropriately qualified physician or other health care professional. The responsibility for the protection of research subjects must always rest with the physician or other health care professional and never the research subjects, even though they have given consent.    7. Medical research involving a disadvantaged or vulnerable population or community is only justified if the research is responsive to the health needs and priorities of this population or community and if there is a reasonable likelihood that this population or community stands to benefit from the results of the research.    8. Every medical research study involving human subjects must be preceded by careful assessment of predictable risks and burdens to the individuals and communities involved in the research in comparison with foreseeable benefits to them and to other individuals or communities affected by the condition under investigation.    9. Every clinical trial must be registered in a publicly accessible database before recruitment of the first subject.    10. Physicians may not participate in a research study involving human subjects unless they are confident that the risks involved have been adequately assessed and can be satisfactorily managed. Physicians must immediately stop a study when the risks are found to outweigh the potential benefits or when there is conclusive proof of positive and beneficial results.    11. Medical research involving human subjects may only be conducted if the importance of the objective outweighs the inherent risks and burdens to the research subjects.    12. Participation by competent individuals as subjects in medical research must be voluntary. Although it may be appropriate to consult family members or community leaders, no competent individual may be enrolled in a research study unless he or she freely agrees.    13. Every precaution must be taken to protect the privacy of research subjects and the confidentiality of their personal information and to minimize the impact of the study on their physical, mental and social integrity.    14. In medical research involving competent human subjects, each potential subject must be adequately informed of the aims, methods, sources of funding, any possible conflicts of interest, institutional affiliations of the researcher, the anticipated benefits and potential risks of the study and the discomfort it may entail, and any other relevant aspects of the study. The potential subject must be informed of the right to refuse to participate in the study or to withdraw consent to participate at any time without reprisal. Special attention should be given to the specific information needs of individual potential subjects as well as to the methods used to deliver the information. After ensuring that the potential subject has understood the information, the physician or another appropriately qualified individual must then seek the potential subject's freely-given informed consent, preferably in writing. If the consent cannot be expressed in writing, the non-written consent must be formally documented and witnessed.    15. For medical research using identifiable human material or data, physicians must normally seek consent for the collection, analysis, storage and/or reuse. There may be situations where consent would be impossible or impractical to obtain for such research or would pose a threat to the validity of the research. In such situations the research may be done only after consideration and approval of a research ethics committee.    16. When seeking informed consent for participation in a research study the physician should be particularly cautious if the potential subject is in a dependent relationship with the physician or may consent under duress. In such situations the informed consent should be sought by an appropriately qualified individual who is completely independent of this relationship.    17. For a potential research subject who is incompetent, the physician must seek informed consent from the legally authorized representative. These individuals must not be included in a research study that has no likelihood of benefit for them unless it is intended to promote the health of the population represented by the potential subject, the research cannot instead be performed with competent persons, and the research entails only minimal risk and minimal burden.    18. When a potential research subject who is deemed incompetent is able to give assent to decisions about participation in research, the physician must seek that assent in addition to the consent of the legally authorized representative. The potential subject's dissent should be respected.    19. Research involving subjects who are physically or mentally incapable of giving consent, for example, unconscious patients, may be done only if the physical or mental condition that prevents giving informed consent is a necessary characteristic of the research population. In such circumstances the physician should seek informed consent from the legally authorized representative. If no such representative is available and if the research cannot be delayed, the study may proceed without informed consent provided that the specific reasons for involving subjects with a condition that renders them unable to give informed consent have been stated in the research protocol and the study has been approved by a research ethics committee. Consent to remain in the research should be obtained as soon as possible from the subject or a legally authorized representative.    20. Authors, editors and publishers all have ethical obligations with regard to the publication of the results of research. Authors have a duty to make publicly available the results of their research on human subjects and are accountable for the completeness and accuracy of their reports. They should adhere to accepted guidelines for ethical reporting. Negative and inconclusive as well as positive results should be published or otherwise made publicly available. Sources of funding, institutional affiliations and conflicts of interest should be declared in the publication. Reports of research not in accordance with the principles of this Declaration should not be accepted for publication. 2. ADDITIONAL PRINCIPLES FOR MEDICAL RESEARCH COMBINED WITH MEDICAL CARE    1. The physician may combine medical research with medical care only to the extent that the research is justified by its potential preventive, diagnostic or therapeutic value and if the physician has good reason to believe that participation in the research study will not adversely affect the health of the patients who serve as research subjects.    2. The benefits, risks, burdens and effectiveness of a new intervention must be tested against those of the best current proven intervention, except in the following circumstances:    - The use of placebo, or no treatment, is acceptable in studies where no current proven intervention exists; or    - Where for compelling and scientifically sound methodological reasons the use of placebo is necessary to determine the efficacy or safety of an intervention and the patients who receive placebo or no treatment will not be subject to any risk of serious or irreversible harm. Extreme care must be taken to avoid abuse of this option.    1. At the conclusion of the study, patients entered into the study are entitled to be informed about the outcome of the study and to share any benefits that result from it, for example, access to interventions identified as beneficial in the study or to other appropriate care or benefits.    2. The physician must fully inform the patient which aspects of the care are related to the research. The refusal of a patient to participate in a study or the patient's decision to withdraw from the study must never interfere with the patient-physician relationship.    3. In the treatment of a patient, where proven interventions do not exist or have been ineffective, the physician, after seeking expert advice, with informed consent from the patient or a legally authorized representative, may use an unproven intervention if in the physician's judgement it offers hope of saving life, re-establishing health or alleviating suffering. Where possible, this intervention should be made the object of research, designed to evaluate its safety and efficacy. In all cases, new information should be recorded and, where appropriate, made publicly available.   22.10.2008 |
